# Does *Chlorella* Supplementation Improve Adiposity, Metabolic Dysfunction, and Oxidative Stress in Individuals With Excess Weight? A Systematic Review and Meta‐Analysis

**DOI:** 10.1002/fsn3.71715

**Published:** 2026-04-22

**Authors:** Ali Jafari, Helia Mardani, Mahsa Mahmoudinezhad, Mohammad Amin Karimi, Vali Musazadeh, Mohammad Sharifi

**Affiliations:** ^1^ Student Research Committee, Department of Community Nutrition, Faculty of Nutrition Sciences and Food Technology, National Nutrition and Food Technology Research Institute Shahid Beheshti University of Medical Sciences Tehran Iran; ^2^ Systematic Review and Meta‐Analysis Expert Group (SRMEG) Universal Scientific Education and Research Network (USERN) Tehran Iran; ^3^ Students' Scientific Research Center (SSRC) Tehran University of Medical Sciences Tehran Iran; ^4^ Food and Beverages Safety Research Center Urmia University of Medical Sciences Urmia Iran; ^5^ Student Research Committee Urmia University of Medical Sciences Urmia Iran; ^6^ School of Medicine Shahid Beheshti University of Medical Sciences Tehran Iran; ^7^ Student Research Committee, School of Public Health Iran University of Medical Sciences Tehran Iran; ^8^ Department of Nutrition, School of Public Health Iran University of Medical Sciences Tehran Iran; ^9^ Department of Nutrition, Food Sciences and Clinical Biochemistry, School of Medicine, Social Determinants of Health Research Center Gonabad University of Medical Science Gonabad Iran

**Keywords:** anthropometric measures, *Chlorella*, glycemic profile, lipid profile, obesity, oxidative stress parameters

## Abstract

This systematic review and meta‐analysis was conducted to investigate the effects of *Chlorella* supplementation on cardiometabolic health indicators in individuals with overweight or obesity. A systematic search of PubMed, Scopus, Embase, Web of Science, and the Cochrane Central Register of Controlled Trials was conducted up to November 2024. Eighteen studies published between 2012 and 2024 were included, involving a total of 717 participants. The duration of the trials ranged from 1 to 12 weeks, with daily *Chlorella* doses varying from 135 to 15,000 mg/day. *Chlorella* supplementation significantly reduced body fat percentage (WMD = −0.72%, 95% CI: −1.11 to −0.34), body mass index (WMD = −0.35 kg/m^2^, 95% CI: −0.55 to −0.14), weight (WMD = −1.41 kg; 95% CI: −2.19 to −0.64), Waist‐Hip Ratio (WMD = −0.01, 95% CI: −0.02 to −0.01), Homeostatic Model Assessment for Insulin Resistance (WMD = −0.16 units, 95% CI: −0.29 to −0.04), insulin levels (WMD = −1.00 μU/mL, 95% CI: −1.34 to −0.66), low‐density lipoprotein cholesterol (WMD = −5.73 mg/dL, 95% CI: −8.77 to −2.70), total cholesterol (WMD = −6.62 mg/dL, 95% CI: −10.18 to −3.05), triglycerides (WMD = −3.24 mg/dL, 95% CI: −6.25 to −0.23), alanine aminotransferase (WMD = −5.22 IU/L, 95% CI: −8.78 to −1.65), aspartate aminotransferase (WMD = −3.45 IU/L, 95% CI: −5.00 to −1.90), and leptin (WMD = −0.66 ng/mL, 95% CI: −1.24 to −0.08). Additionally, there were significant increases in catalase activity (WMD = 19.15 IU/g Hb, 95% CI: 0.44 to 37.85), and superoxide dismutase levels (WMD = 20.53 U/L, 95% CI: 15.03 to 26.02). *Chlorella* supplementation may benefit anthropometric and cardiometabolic outcomes in adults with overweight or obesity. However, the overall certainty of evidence was low to very low according to the Grading of Recommendations, Assessment, Development, and Evaluation (GRADE) framework, limiting confidence in these findings. Moreover, most included trials were conducted in Iran, which may restrict generalizability. Further high‐quality, well‐powered randomized trials in diverse populations are needed to confirm these effects.

## Graphical abstract

*Chlorella* supplementation significantly improves adiposity, metabolic parameters, and oxidative stress markers in individuals with overweight or obesity. This systematic review and meta‐analysis of 18 studies (*n* = 717) demonstrates beneficial effects on body fat percentage, BMI, weight, waist‐hip ratio, insulin resistance, lipid profile, liver enzymes, leptin, and antioxidant activity. Findings support the potential of *Chlorella* as a complementary intervention for enhancing cardiometabolic health in overweight and obese populations.

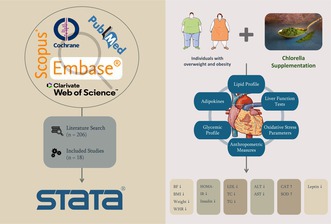

**Abbreviations**
ALP: alkaline phosphatase
ALT: alanine aminotransferase
AST: aspartate aminotransferase
BF%: body fat percentage
BMI: body mass index
BMR: basal metabolic rate
*C. vulgaris*
: *Chlorella vulgaris*
CAT: catalase
CENTRAL: cochrane central register of controlled trials
CVD: cardiovascular diseases
DB: double‐blinded
FBG: fasting blood glucose
GPx: glutathione peroxidase
GRADE: grading of recommendations, assessment, development, and evaluation
HC: hip circumference
HDL‐C: high‐density lipoprotein cholesterol
HIIT: high‐intensity interval training
HOMA‐IR: homeostasis model assessment of insulin resistance
LDL‐C: low‐density lipoprotein cholesterol
MDA: malondialdehyde
NAFLD: nonalcoholic fatty liver disease
PC: placebo‐controlled
PRISMA: preferred reporting items for systematic reviews and meta‐analyses
PROSPERO: international prospective register of systematic reviews
RCT: randomized controlled trial
ROS: reactive oxygen species
SD: standard deviation
SOD: superoxide dismutase
T2DM: type 2 diabetes mellitus
TAC: total antioxidant capacity
TC: total cholesterol
TG: triglycerides
WC: waist circumference
WHR: waist‐to‐hip ratio

## 1 Introduction

Obesity and overweight are modifiable metabolic risk factors characterized by a prolonged positive energy balance, which contributes to the excessive accumulation of adipose tissue (Singh‐Manoux et al. 2018). According to the World Health Organization (WHO), in adults, overweight is defined as a body mass index (BMI) of 25.0–29.9 kg/m^2^, while obesity is defined as a BMI ≥ 30 kg/m^2^. Recent WHO estimates indicate that in 2022, approximately 43% of adults aged 18 years and older were living with overweight and 16% with obesity, underscoring the substantial global burden of these metabolic conditions (Organization WH 2025). Obesity is closely linked to a diminished quality of life. It significantly elevates the risk of numerous chronic conditions, including cardiovascular diseases (CVD), type 2 diabetes (T2DM), osteoarthritis, and certain cancers, often leading to premature mortality (Safaei et al. 2021; Khani et al. 2025; Zhang et al. 2024; Hutten et al. 2024). The pathophysiology of obesity is multifactorial, involving complex genetic, behavioral, and primarily environmental factors such as sedentary lifestyles and high‐calorie diets (El‐Sayed Moustafa and Froguel 2013).

Given the serious health risks linked to excess weight, implementing effective and compassionate interventions remains a public health priority. Alongside dietary changes, physical activity, and behavioral therapies, pharmacological treatments have been employed to support individuals with overweight and obesity in managing their weight and improving their health outcomes (Kahan 2016; Kheniser et al. 2021). However, most available medications are synthetic, often costly, and associated with adverse side effects (Arterburn et al. 2020; Liu et al. 2025). Despite substantial investment in the development of pharmacotherapies, only a limited number have received regulatory approval. This approach has driven growing interest in complementary and alternative approaches. Among these, herbal medicines and their derivatives have gained attention for their potential to address obesity related concerns (Shaik Mohamed Sayed et al. 2023; Hasani‐Ranjbar et al. 2013; Rucker et al. 2007). These compounds exert anti‐obesity effects through several mechanisms, such as suppressing appetite and reducing caloric intake, enhancing thermogenesis and metabolic activity, inhibiting pancreatic lipase to limit fat absorption, promoting lipolysis, and inhibiting lipogenesis (Apovian et al. 2015; Kazemipoor et al. 2014). Several studies have explored the impact of herbal formulations on metabolic parameters, highlighting their potential in managing obesity‐related complications (Jafari, Mardani, Faghfouri, AhmadianMoghaddam, et al. 2025; Jafari, Mardani, Faghfouri, Fashtali, et al. 2025). Among these compounds, *Chlorella* has attracted growing scientific attention.

*Chlorella*, a nutrient‐dense green microalga, is characterized by a high content of dietary fiber, carotenoids, chlorophyll, essential vitamins, minerals, and long‐chain polyunsaturated fatty acids. These bioactive constituents underpin its diverse spectrum of biological activities and potential therapeutic applications (Panahi, Pishgoo, et al. 2012; Han et al. 2015; Bito et al. 2020a). This unicellular alga demonstrates a range of bioactive effects that contribute to metabolic health, including antioxidant, anti‐inflammatory, and cardiometabolic properties (Barghchi et al. 2023a; Jafari et al. 2026). Through mechanisms such as improving insulin sensitivity, reducing hepatic lipid accumulation, and alleviating oxidative stress, it has been proposed as a complementary therapeutic approach for the management of dyslipidemia, hyperglycemia, and obesity (Bito et al. 2020a; Ebrahimi‐Mameghani et al. 2017; Barghchi et al. 2023b; Mendes et al. 2024). However, findings from existing studies are mixed, with some reporting beneficial outcomes on weight‐related parameters and metabolic biomarkers, while others show inconclusive or conflicting results (Regueiras et al. 2021; Sanayei, Kalejahi, et al. 2022).

Despite the growing body of literature investigating the effects of *Chlorella* supplementation on metabolic health, prior meta‐analyses in this area exhibit several methodological limitations that warrant the need for an updated and more rigorous synthesis. The widely cited meta‐analysis by Fallah et al. (Fallah et al. 2018) primarily examined cardiovascular risk factors but overlooked numerous relevant trials that should have been included in the analysis (Okuda et al. 1975; Panahi, Ghamarchehreh, et al. 2012; Panahi, Tavana, et al. 2012; Ebrahimi‐Mameghani et al. 2013; Aliashrafi et al. 2014; Umemoto and Otsuki 2014). Moreover, several included studies inaccurately categorized interventions, incorporating trials where the intervention groups received confounding agents such as vitamin E (Ebrahimi‐Mameghani et al. 2017, 2014b) or atorvastatin (Panahi, Pishgoo, et al. 2012), thus undermining the ability to attribute observed effects solely to *Chlorella*. Additional inaccuracies, including misreporting of sample sizes (Inoue et al. 1995) and erroneous classification of study durations (Nakano et al. 2010), further compromised the statistical validity of their conclusions. Since the publication of Fallah et al.'s study, a substantial number of randomized controlled trials have emerged (Esmaieli et al. 2018; Haidari et al. 2018; Govahi et al. 2019; Karbalamahdi et al. 2019; Shafeie et al. 2019; Vakili et al. 2019; Samadi et al. 2020; Chiu et al. 2021; Hosseini et al. 2021; Sanayei et al. 2021; Tofighi et al. 2021, 2024; Sanayei, Hajizadeh‐Sharafabad, et al. 2022; Amiri Mandoulakani et al. 2023; Sandgruber et al. 2023; Delfan, Radkia, et al. 2024; Delfan, Behzadi, et al. 2024), underscoring the necessity for an updated search strategy to reflect the most current evidence. Yarmohammadi et al. (Yarmohammadi et al. 2021a) concentrated narrowly on liver function biomarkers (AST, ALT, ALP) and reported inconsistent findings across different populations, whereas a broader assessment of cardiometabolic factors remains lacking. Sanayei et al. (Sanayei, Kalejahi, et al. 2022) highlighted insufficient statistical power in existing studies, limiting the reliability of their conclusions regarding metabolic outcomes. Furthermore, Sherafati et al. (Sherafati et al. 2022) focused predominantly on lipid profiles and applied dose–response analyses only within a restricted dosage range (≤ 1500 mg/day), limiting exploration of higher *Chlorella* dosages and their potential effects.

This systematic review critically examines the potential impact of *Chlorella* supplementation on individuals with overweight and obesity, focusing on a comprehensive range of metabolic outcomes, including glycemic profiles, anthropometric indices, lipid parameters, liver function biomarkers, and oxidative stress levels. To address the limitations of previous meta‐analyses, we incorporated a larger number of randomized controlled trials, rectified prior misclassifications, applied dose–response meta‐analysis across a broader dosage spectrum, and systematically assessed the certainty of evidence using the Grading of Recommendations, Assessment, Development, and Evaluation (GRADE) approach. Unlike earlier reviews, we imposed no language restrictions during study selection, enabling the inclusion of a wider and more diverse body of research. We also employed advanced statistical techniques to explore how variations in dosage and intervention duration influence these health indicators. Collectively, these methodological improvements enhance the precision, comprehensiveness, and clinical relevance of our findings, offering clear, evidence‐based insights to support personalized care strategies and inform public health interventions aimed at improving metabolic health and quality of life among individuals affected by excess weight.

## 2 Methods

### 2.1 Protocol and Registration

The present study followed the guidelines outlined in the Preferred Reporting Items for Systematic Reviews and Meta‐Analyses (PRISMA) 2020 guidelines (Page et al. 2021). This systematic review and meta‐analysis was registered in the International Prospective Register of Systematic Reviews (PROSPERO) with registration number CRD420251052016 obtained to uphold transparency and commitment to the predefined methodological framework.

### 2.2 Search Strategy and Study Selection

A thorough and methodological literature search was conducted across multiple electronic databases, including PubMed, Web of Science, Scopus, Embase, and the Cochrane Central Register of Controlled Trials (CENTRAL), encompassing studies published up to November 2024. Additional searches were carried out through Google Scholar, and reference lists from key articles, systematic reviews, and high‐impact journals were manually reviewed to ensure the inclusion of all relevant evidence. Gray literature sources such as ProQuest Dissertations and Theses, Open Gray, and major conference proceedings were also examined to capture unpublished data. This comprehensive search strategy aimed to identify trials assessing the effects of *Chlorella* supplementation on anthropometric parameters, glycemic control, lipid profiles, hepatic enzymes, oxidative stress biomarkers, and leptin concentrations. Database‐specific strategies were tailored using a combination of controlled vocabulary terms from MeSH and EMTREE alongside relevant keywords such as “*Chlorella*,” “microalgae,” “ *C. vulgaris* ,” “ *C. pyrenoidosa* ,” “obesity,” “overweight,” and clinical trial identifiers (Table S1).

### 2.3 Inclusion/Exclusion Criteria and Data Extraction

Eligible studies comprised trials involving adult populations with a BMI of ≥ 25 kg/m^2^, comparing *Chlorella* supplementation to control interventions, regardless of participants' baseline health status. No limitations were applied concerning language, publication status, or duration of the intervention. Studies were excluded if they involved animal subjects, incorporated additional concurrent interventions, or failed to provide sufficient outcome data.

Two independent reviewers (A.J. and H.M.) conducted the initial screening of titles and abstracts to identify potentially relevant studies, followed by a full‐text assessment of those meeting preliminary inclusion criteria. Discrepancies in study selection were resolved through mutual discussion, involving a third reviewer (V.M.) when consensus could not be reached. Search results were organized and duplicates removed using EndNote X7, and a standardized data extraction form was employed to collect relevant information, including study authorship, year of publication, geographical location, design characteristics, participant details, intervention protocols, and outcome measures.

### 2.4 Methodological Quality and Strength of Evidence Assessment

The methodological rigor of the included trials was assessed using version 2 of the Cochrane Risk of Bias tool (RoB 2), which enables structured evaluation across key domains. Two reviewers (M.M., M.A.K.) independently appraised each study, with disagreements resolved through discussion or, when necessary, consultation with a third reviewer (M.S.). The assessment covered domains such as the generation of the randomization sequence, allocation concealment, blinding practices, handling of incomplete outcome data, selective reporting, and other potential sources of bias.

The GRADE approach was applied to evaluate the strength and reliability of the evidence for each outcome. This framework classifies evidence quality into four levels (high, moderate, low, or very low) based on factors including study limitations, consistency of results, precision, directness of evidence, and risk of publication bias. Two reviewers independently conducted these evaluations, with any differences resolved through consensus or arbitration by a third reviewer (A.J.).

### 2.5 Statistical Analysis

Statistical analyses were conducted using Stata software, version 15.0 (Stata Corp, College Station, TX, USA), to evaluate the potential effects of *Chlorella* supplementation on cardiometabolic health indicators. Data on means, standard deviations (SDs), and sample sizes at baseline and post‐intervention were systematically extracted for outcomes. When essential information was missing, study authors were contacted directly to support a complete and transparent analysis.

To enable comparison across diverse measurement tools, weighted mean differences (WMDs) with 95% confidence intervals (CIs) were calculated (Chandler et al. 2019). A random‐effects meta‐analysis using the DerSimonian and Laird method was applied to account for variability between studies, reflecting scientific rigor and clinical populations' real‐world diversity (Borenstein et al. 2010). Heterogeneity was assessed using the *I* ^2^ statistic, with values categorized to represent varying degrees of inconsistency (Higgins and Thompson 2002).

Subgroup analyses were conducted based on baseline BMI, *Chlorella* dosage, intervention duration, sex, intervention approach, *Chlorella* species, intervention type, country, baseline health condition, participant age, control approach, and sample size. Meta‐regression analyses were performed to assess the influence of *Chlorella* dosage and intervention duration on the outcomes (Orsini et al. 2006). A non‐linear dose–response model was also employed to assess the relationship between different supplementation levels and observed health effects, aiming to inform evidence‐based guidance on optimal dosing and duration (Xu and Doi 2018). To evaluate the robustness of the findings, influence analyses were performed, and potential publication bias was assessed using both funnel plot visualizations and formal statistical tests (Egger's and Begg's), ensuring a balanced and ethically responsible interpretation of the evidence (Egger et al. 1997).

## 3 Results

### 3.1 Study Selection

**FIGURE 1:**
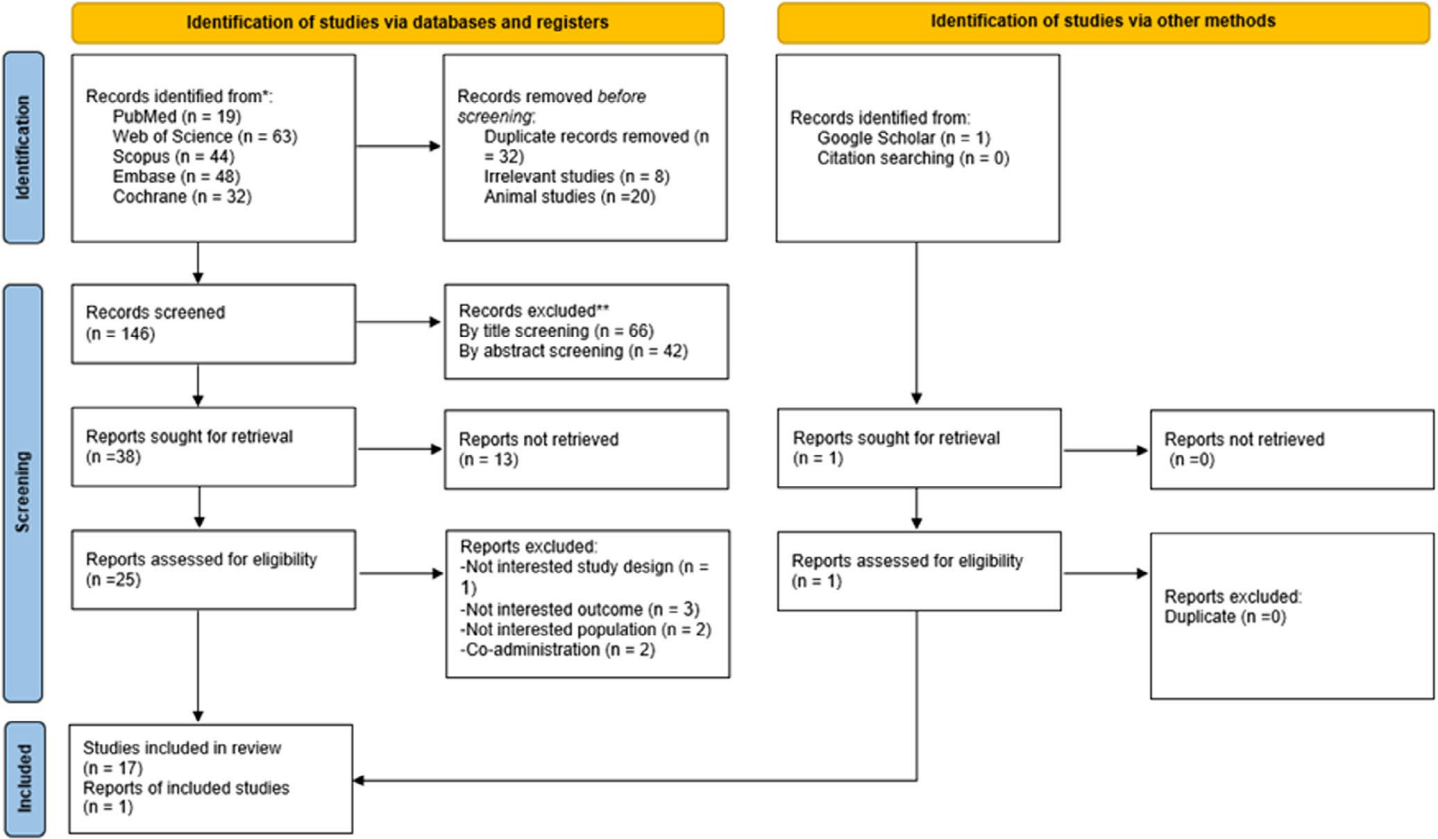
Flowchart of study selection for inclusion trials in the systematic review.

A systematic literature search identified 206 records from PubMed (*n* = 19), Web of Science (*n* = 63), Scopus (*n* = 44), Embase (*n* = 48), and Cochrane (*n* = 32). After removing duplicates (*n* = 32), irrelevant studies (*n* = 8), and animal studies (*n* = 20), 146 articles underwent title and abstract screening. Of these, 108 were excluded due to mismatched objectives, populations, or outcomes. Full‐text retrieval was attempted for the remaining 38 studies; however, 13 could not be accessed, leaving 25 reports for eligibility assessment. During the full‐text review, eight studies were excluded for reasons including irrelevant study design (*n* = 1), not interested outcomes (*n* = 3), not interested populations (*n* = 2), and co‐administration of interventions (*n* = 2). Additionally, one study was obtained from Google Scholar. Ultimately, 18 studies met the inclusion criteria and were included for qualitative and quantitative synthesis (Panahi, Tavana, et al. 2012; Esmaieli et al. 2018; Govahi et al. 2019; Karbalamahdi et al. 2019; Shafeie et al. 2019; Vakili et al. 2019; Samadi et al. 2020; Chiu et al. 2021; Hosseini et al. 2021; Sanayei et al. 2021; Tofighi et al. 2021, 2024; Sanayei, Hajizadeh‐Sharafabad, et al. 2022; Amiri Mandoulakani et al. 2023; Sandgruber et al. 2023; Delfan, Radkia, et al. 2024; Delfan, Behzadi, et al. 2024; Chitsaz et al. 2016). The PRISMA flow diagram illustrates the study selection process (Figure 1).

### 3.2 Study Characteristics

**FIGURE 2:**
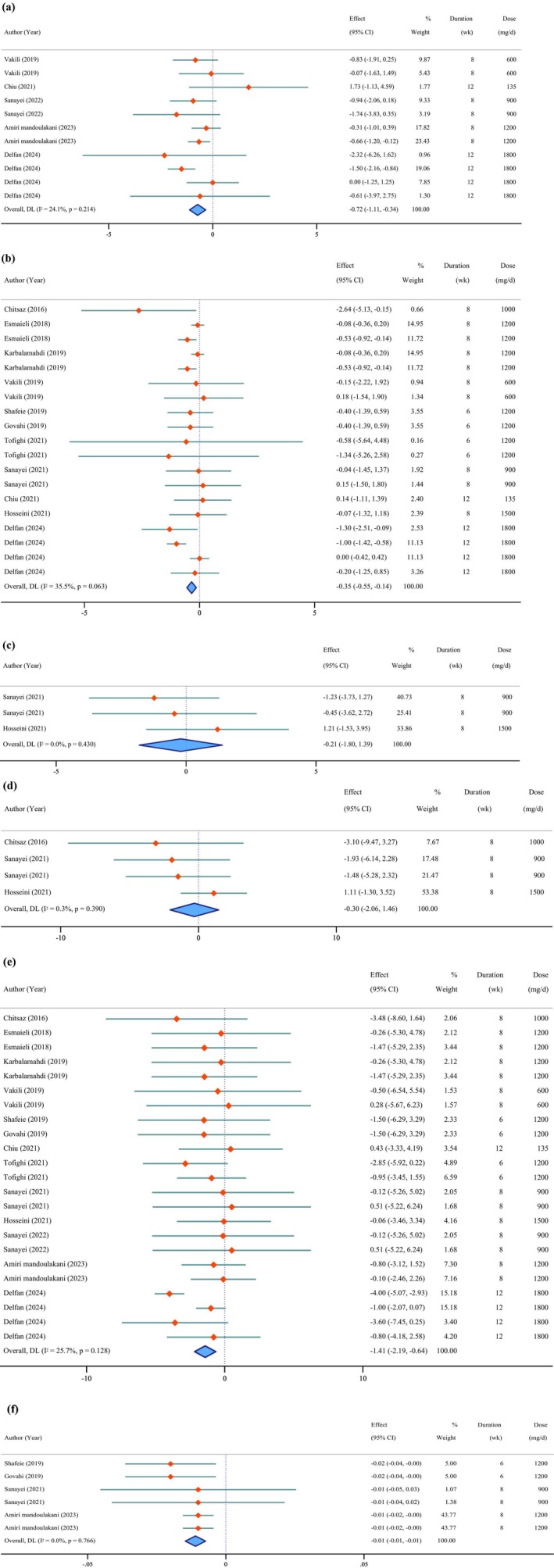
Forest plot of the effects of *Chlorella* supplement on anthropometric measures (a: Body fat percentage, b: Body mass index, c: Hip circumference, d: Waist circumference, e: Weight, f: Waist‐to‐hip ratio).

**FIGURE 3:**
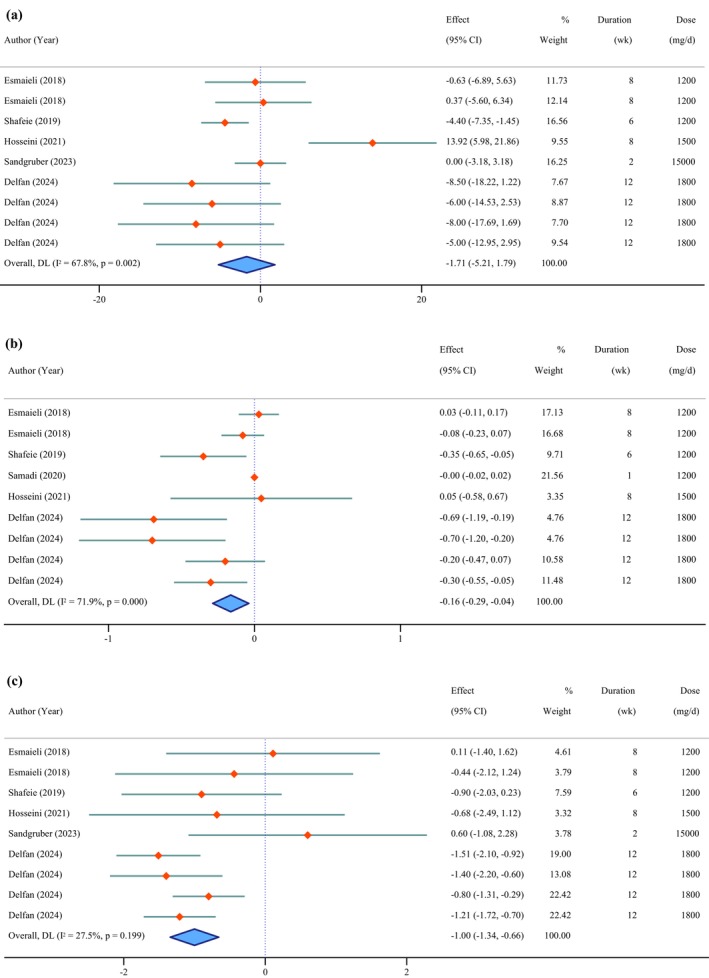
Forest plot of the effects of *Chlorella* supplement on glycemic profile (a: Fasting blood glucose, b: Homeostatic model assessment for insulin resistance, c: Insulin).

**FIGURE 4:**
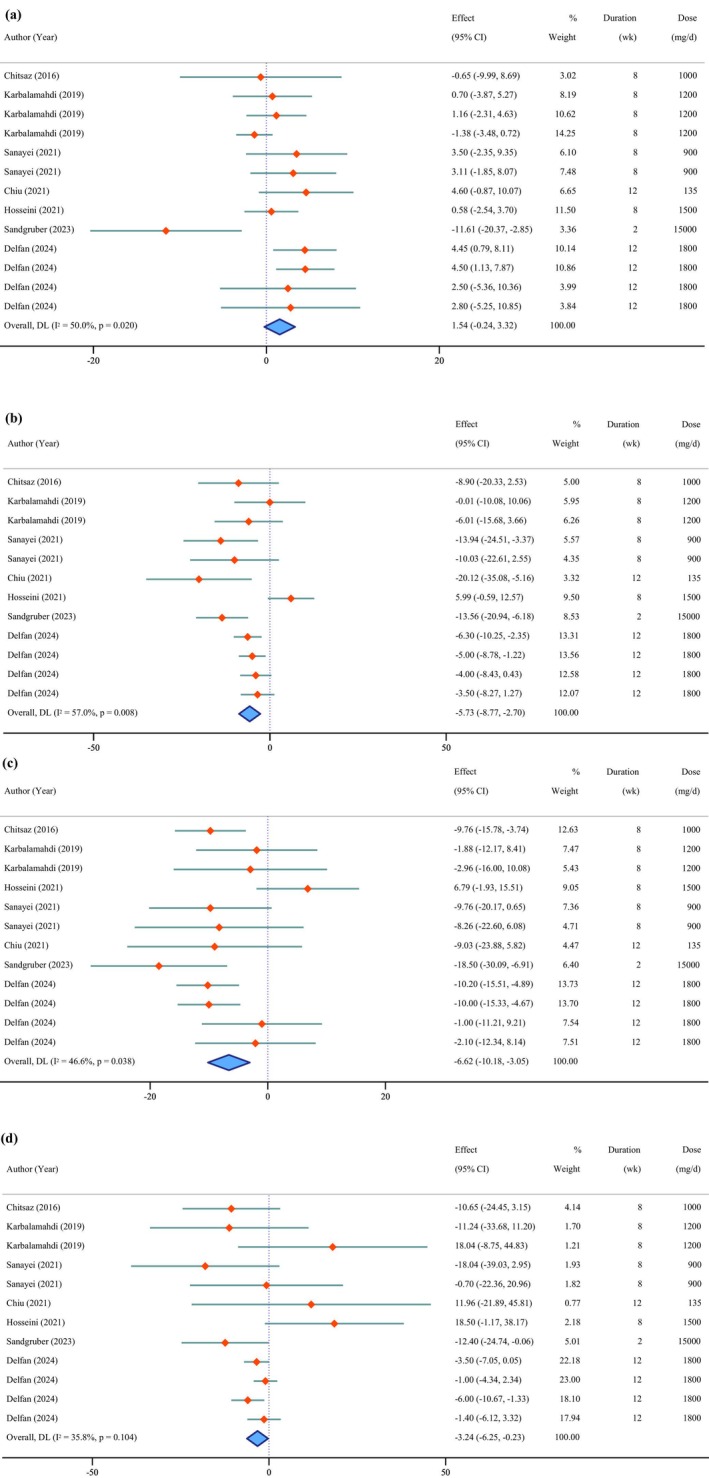
Forest plot of the effects of *Chlorella* supplement on lipid profile (a: High‐density lipoprotein cholesterol, b: Low‐density lipoprotein cholesterol, c: Total cholesterol, d: Triglycerides).

**FIGURE 5:**
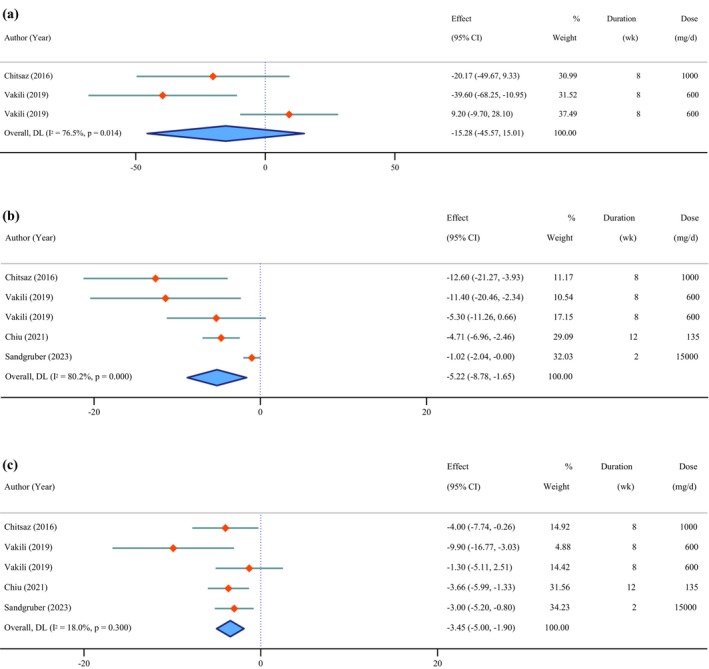
Forest plot of the effects of *Chlorella* supplement on liver function tests (a: Alkaline phosphatase, b: Alanine aminotransferase, c: Aspartate aminotransferase).

**FIGURE 6:**
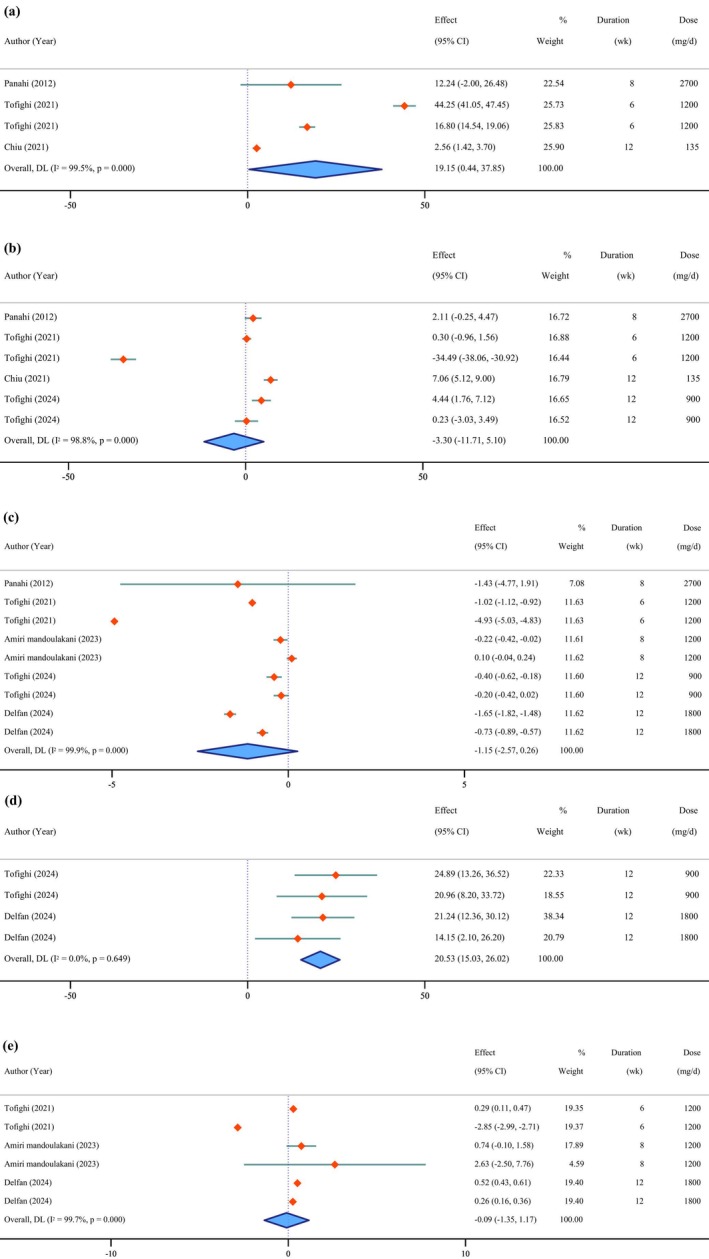
Forest plot of the effects of *Chlorella* supplement on oxidative stress parameters (a: Catalase, b: Glutathione peroxidase, c: Malondialdehyde, d: Superoxide dismutase, e: Total antioxidant capacity).

**FIGURE 7:**
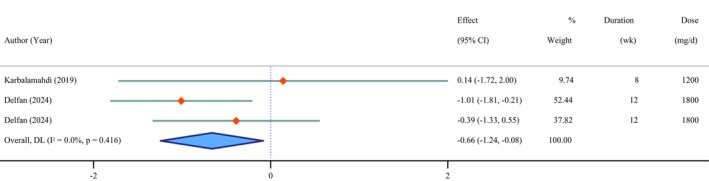
Forest plot of the effects of *Chlorella* supplement on leptin.

The characteristics of the 18 included studies are summarized in Table 1. The WMDs and 95% CI for changes in body fat percentage (BF%), BMI, hip circumference (HC), waist circumference (WC), weight, waist‐to‐hip ratio (WHR), fasting blood glucose (FBG), homeostatic model assessment for insulin resistance (HOMA‐IR), insulin, high‐density lipoprotein cholesterol (HDL‐C), low‐density lipoprotein cholesterol (LDL‐C), total cholesterol (TC), triglycerides (TG), alkaline phosphatase (ALP), alanine aminotransferase (ALT), aspartate aminotransferase (AST), catalase (CAT), glutathione peroxidase (GPx), malondialdehyde (MDA), superoxide dismutase (SOD), total antioxidant capacity (TAC), and leptin are demonstrated in Figures 2, 3, 4, 5, 6, 7. Moreover, funnel plots for assessing publication bias are provided in Figure 1.

**TABLE 1:** Characteristics of included studies in the meta‐analysis.

Author. (Ref.)	Year	Country	Study design	Health condition	Sex	Sample size (Total)	Sample size (INT/CON)	Dose of supplement (mg/d)	Mean BMI (INT/CON)	Mean age (year) (INT/CON)	Trial duration (week)	Type of supplement (INT/CON)	Outcomes
Panahi, Tavana, et al. (2012)	2012	Iran	RCT	Chronic pulmonary disease	B	57	28/29	2700	28.2/27.45	49.38 ± 2.84/52.42 ± 2.88	8	*C. vulgaris* extract + standard anti‐asthma/COPD treatment/standard anti‐asthma/COPD treatment	CAT, GPX & MDA
Chitsaz et al. (2016)	2016	Iran	RCT	NAFLD	B	41	21/20	1000	29.44 ± 3.69/27.03 ± 1.68	43 ± 7.33/42 ± 8.33	8	Tablet: *C. vulgaris* /Usual care	BMI, WC, weight, HDL‐C, LDL‐C, TC, TG, ALP, ALT & AST
Esmaieli et al. (2018)	2018	Iran	R, DB	Obese	F	32	16/16	1200	30.89 ± 0.34/30.48 ± 0.43	30–45/30–45	8	Tablet: *C. vulgaris* /No supplement & *C. vulgaris* + training/Training	BMI, weight, FBG, insulin & HOMA‐IR
Govahi et al. (2019)	2019	Iran	R, PC	Overweight	M	20	10/10	1200	27.8 ± 1.8/28.2 ± 1.8	22.8 ± 2.2/22.8 ± 2.2	6	Capsule: *C. vulgaris* + exercise/Placebo (sucrose) + exercise	BMI, weight & WHR
Karbalamahdi et al. (2019)	2019	Iran	RCT	Obese	F	32	16/16	1200	30.89 ± 0.34/30.48 ± 0.43 & 31.08 ± 0.63/30.55 ± 0.45	26 ± 4/25.50 ± 3.66 & 28.5 ± 1.77/25 ± 4.17	8	Pill: *C. vulgaris* / No supplement & *C. vulgaris* + aerobic training/Aerobic training	BMI, weight, HDL‐C, LDL‐C, TC, TG & leptin
Shafeie et al. (2019)	2019	Iran	R, PC	Overweight	M	20	10/10	1200	27.8 ± 1.8/28.2 ± 1.8	22.78 ± 2.33/22.78 ± 2.33	6	Tablet: *C. vulgaris* + HIIT/Placebo (sucrose) + HIIT	BMI, weight, WHR, FBG, insulin & HOMA‐IR
Vakili et al. (2019)	2019	Iran	RCT	T2DM	F	40	20/20	600	30.19 ± 4.49/28.73 ± 2.58 & 29.80 ± 2.260/30.26 ± 3.82	56 ± 3.82/55.60 ± 4.22 & 55 ± 3.01/56.80 ± 4.21	8	*C. vulgaris* /No supplement & *C. vulgaris* + endurance training/Endurance training	BF%, BMI, weight, ALP, ALT & AST
Samadi et al. (2020)	2020	Iran	R, PC	Overweight	M	20	10/10	1200	27.19 ± 1.85/28.51 ± 1.43	22.8 ± 2.2/22.8 ± 2.2	1	Tablet: *C. vulgaris* /Placebo (dextrose)	HOMA‐IR
Chiu et al. (2021)	2021	Taiwan	R, PC, DB	Healthy	B	44	21/23	135	25.02 ± 2.88/24.53 ± 3.77	40–75/40–75	12	Water extract: *Chlorella pyrenoidosa* /Placebo (essence of mushroom, algae water, caramel)	BF%, BMI, weight, GPX, CAT, HDL‐C, LDL‐C, TC, TG, ALT & AST
Hosseini et al. (2021)	2021	Iran	R, PC, DB	T2DM	B	75	36/39	1500	27.94 ± 5.12/26.73 ± 3.43	54.75 ± 5.98/55 ± 6.92	8	Capsule: *C. vulgaris* /Placebo (starch)	BMI, HC, WC, weight, FBG, HOMA‐IR, insulin, HDL‐C, LDL‐C, TC & TG
Sanayei et al. (2021)	2021	Iran	R, PC, DB	Overweight & obese	F	46	24/22	900	29.51 ± 2.68/28.66 ± 2.80 & 30.97 ± 3.26/29.98 ± 2.90	27.08 ± 4.29/29.27 ± 5.12 & 26.83 ± 5.82/29.09 ± 4.7	8	Capsule: *C. vulgaris* /placebo (corn starch) & *C. vulgaris* + HIIT/Placebo (corn starch) + HIIT	BMI, HC, WC, weight, WHR, HDL‐C, LDL‐C, TC & TG
Tofighi et al. (2021)	2021	Iran	R, PC	Inactive obese	M	40	20/20	1200	32.76 ± 5.56/31.6 ± 4.54	22.56 ± 1.98/23.74 ± 9.54	6	Capsule: *C. vulgaris* /Placebo (sucrose) & *C. vulgaris* + exercise/Placebo (sucrose) + exercise	BMI, weight, CAT, GPX, MDA & TAC
Sanayei, Hajizadeh‐Sharafabad, et al. (2022)	2022	Iran	R, PC, DB	Obese & overweight	F	46	24/22	900	25–35/25–35	18–35/18–35	8	Capsule: *C. vulgaris* /Placebo (corn starch powder) & *C. vulgaris* + HIIT/Placebo (corn starch powder) + HIIT	BF% & weight
Amiri Mandoulakani et al. (2023)	2023	Iran	Controlled trial	Obese & overweight	F	40	20/20	1200	25 ≤/25 ≤	40–65/40–65	8	Tablet: *Chlorella*/Placebo (sucrose) & *Chlorella* + HIIT/Placebo (sucrose) + HIIT	BF%, weight, WHR, MDA & TAC
Sandgruber et al. (2023)	2023	Germany	RCT	Healthy	B	36	19/17	15,000	≤ 30/≤ 30	20–35/20–35	2	NR: *Chlorella pyrenoidosa* + smoothie (banana, pineapple, kale, mango, dates, avocado, lime juice, wheat grass & mint) + menu plan (MoKaRi)/Menu plan (MoKari)	HDL‐C, LDL‐C, TC, TG, FBG, insulin, ALT & AST
Delfan, Radkia, et al. (2024)	2024	Iran	R, PC, DB	Obese	M	44	22/22	1800	32.6 ± 2/32.3 ± 1.1	23–35/23–35	12	Capsule: *C. vulgaris* /Placebo (flour) & *C. vulgaris* + IRT/Placebo (flour) + IRT	BF%, BMI, weight, FBG, HOMA‐IR, insulin, HDL‐C, LDL‐C, TC, TG, MDA, SOD & TAC
Delfan, Behzadi, et al. (2024)	2024	Iran	R, PC, DB	Obese	M	44	22/22	1800	32 ± 2.0/32 ± 1.5	23–35/23–35	12	Capsule: *C. vulgaris* /Placebo (flour) & *C. vulgaris* + IRT/Placebo (flour) + IRT	BF%, BMI, weight, FBG, HOMA‐IR, insulin, HDL‐C, LDL‐C, TC, TG, & leptin
Tofighi et al. (2024)	2024	Iran	R, PC	Overweight & obese	M	40	20/20	900	32.74 ± 3.45/33.65 ± 1.85 & 32.85 ± 2.65/33.85 ± 1.75	29.7 ± 4.1/30.2 ± 4.8 & 30.5 ± 3.9/31.8 ± 3.1	12	Capsule: *C. vulgaris* /Placebo (corn starch) & *C. vulgaris* + functional exercise/Placebo (corn starch) + functional exercise	GPX, MDA & SOD

Abbreviations: ALT, alanine aminotransferase; ALP, alkaline phosphatase; AST, aspartate aminotransferase; B, both; BF%, body fat percentage; BMI, body mass index; CAT, catalase; *C. vulgaris* , *Chlorella vulgaris* ; CON, control group; COPD, chronic obstructive pulmonary disease; DB, double‐blinded; F, female; FBG, fasting blood glucose; GPX, glutathione peroxidase; HC, hip circumference; HDL‐C, high‐density lipoprotein cholesterol; HIIT, high‐intensity interval training; HOMA‐IR, homeostatic model assessment for insulin resistance; INT, intervention group; IRT, interval resistance training; LDL‐C, low‐density lipoprotein cholesterol; M, male; MDA, malondialdehyde; NAFLD, nonalcoholic fatty liver disease; PC, placebo‐controlled; R, randomized; RCT, randomized controlled trial; Ref, reference; SOD, superoxide dismutase; T2DM, type 2 diabetes mellitus; TAC, total antioxidant capacity; TC, total cholesterol; TG, triglycerides; WC, waist circumference; WHR, waist‐to‐hip ratio.

The studies were published between 2012 and 2024. The studies were conducted in Iran (Panahi, Tavana, et al. 2012; Esmaieli et al. 2018; Govahi et al. 2019; Karbalamahdi et al. 2019; Shafeie et al. 2019; Vakili et al. 2019; Samadi et al. 2020; Hosseini et al. 2021; Sanayei et al. 2021; Tofighi et al. 2021, 2024; Sanayei, Hajizadeh‐Sharafabad, et al. 2022; Amiri Mandoulakani et al. 2023; Delfan, Radkia, et al. 2024; Delfan, Behzadi, et al. 2024; Chitsaz et al. 2016), Taiwan (Chiu et al. 2021), and Germany (Sandgruber et al. 2023), predominantly focused on individuals with overweight and/or obesity (Esmaieli et al. 2018; Govahi et al. 2019; Karbalamahdi et al. 2019; Shafeie et al. 2019; Samadi et al. 2020; Sanayei et al. 2021; Tofighi et al. 2021, 2024; Sanayei, Hajizadeh‐Sharafabad, et al. 2022; Amiri Mandoulakani et al. 2023; Sandgruber et al. 2023; Delfan, Radkia, et al. 2024; Delfan, Behzadi, et al. 2024), T2DM (Vakili et al. 2019; Hosseini et al. 2021), nonalcoholic fatty liver disease (NAFLD) (Chitsaz et al. 2016), chronic pulmonary disease (Panahi, Tavana, et al. 2012), or healthy individuals (Chiu et al. 2021). The sample sizes ranged from 20 to 75 participants. The trials employed diverse designs, including randomized controlled trials (RCTs), double‐blinded (DB), placebo‐controlled (PC), and crossover methodologies. Intervention durations varied widely, spanning 1 to 12 weeks, though most (Panahi, Tavana, et al. 2012; Esmaieli et al. 2018; Govahi et al. 2019; Karbalamahdi et al. 2019; Shafeie et al. 2019; Vakili et al. 2019; Hosseini et al. 2021; Sanayei et al. 2021; Tofighi et al. 2021; Sanayei, Hajizadeh‐Sharafabad, et al. 2022; Amiri Mandoulakani et al. 2023; Chitsaz et al. 2016) lasted 6–8 weeks. *Chlorella vulgaris* ( *C. vulgaris* ) supplementation dosages ranged from 135 to 2700 mg/day, administered as tablets, capsules, or water extracts. In comparison, one study utilized *Chlorella pyrenoidosa* at 15,000 mg/day combined with a dietary regimen (Sandgruber et al. 2023). Several studies (Esmaieli et al. 2018; Govahi et al. 2019; Karbalamahdi et al. 2019; Shafeie et al. 2019; Vakili et al. 2019; Sanayei et al. 2021; Tofighi et al. 2021, 2024; Sanayei, Hajizadeh‐Sharafabad, et al. 2022; Amiri Mandoulakani et al. 2023; Delfan, Radkia, et al. 2024; Delfan, Behzadi, et al. 2024) also incorporated physical activity regimens such as high‐intensity interval training (HIIT), aerobic exercise, and endurance training.

A total of 717 participants were involved, with 359 in the intervention group and 358 in the control group. The sample sizes for the intervention and control groups across various outcomes were as follows: BF%: *n* = 258 (intervention: 129, control: 129); BMI: *n* = 478 (intervention: 238, control: 240); HC: *n* = 121 (intervention: 60, control: 61); WC: *n* = 162 (intervention: 81, control: 81); weight: *n* = 564 (intervention: 282, control: 282); WHR: *n* = 126 (intervention: 64, control: 62); FBG: *n* = 251 (intervention: 125, control: 125); HOMA‐IR: *n* = 235 (intervention: 116, control: 119); insulin: *n* = 251 (intervention: 125, control: 126); HDL‐C: *n* = 378 (intervention: 189, control: 189); LDL‐C: *n* = 362 (intervention: 181, control: 181); TC: *n* = 362 (intervention: 181, control: 181); TG: *n* = 362 (intervention: 181, control: 181); ALP: *n* = 81 (intervention: 41, control: 40); AST: *n* = 161 (intervention: 81, control: 80); CAT: *n* = 141 (intervention: 69, control: 72); GPx: *n* = 181 (intervention: 89, control: 92); MDA: *n* = 221 (intervention: 110, control: 111); SOD: *n* = 84 (intervention: 42, control: 42); TAC: *n* = 124 (intervention: 62, control: 62); leptin: *n* = 60 (intervention: 30, control: 30).

### 3.3 Qualitative Data Assessment

Based on the RoB 2 tool, most studies were found to have a high risk of bias, particularly in areas such as blinding of outcome assessment and allocation concealment. Overall, the studies (Panahi, Tavana, et al. 2012; Esmaieli et al. 2018; Govahi et al. 2019; Karbalamahdi et al. 2019; Shafeie et al. 2019; Vakili et al. 2019; Samadi et al. 2020; Chiu et al. 2021; Hosseini et al. 2021; Tofighi et al. 2021, 2024; Amiri Mandoulakani et al. 2023; Sandgruber et al. 2023; Delfan, Radkia, et al. 2024; Delfan, Behzadi, et al. 2024; Chitsaz et al. 2016) were rated as poor quality, with only a few (Sanayei et al. 2021; Sanayei, Hajizadeh‐Sharafabad, et al. 2022) demonstrating fair quality (Table 2).

**TABLE 2:** Quality of included studies in the meta‐analysis.

Study, year	Random sequence generation	Allocation concealment	Blinding of participants & personnel	Blinding of outcome assessment	Incomplete outcome data	Selective outcome reporting	Other sources of bias	Overall quality
Panahi, Tavana, et al. (2012)	U	H	H	H	H	L	L	Poor
Chitsaz et al. (2016)	L	H	H	H	L	L	L	Poor
Esmaieli et al. (2018)	H	H	L	H	U	L	L	Poor
Govahi et al. (2019)	L	H	L	H	L	L	L	Poor
Karbalamahdi et al. (2019)	U	H	H	H	U	L	L	Poor
Shafeie et al. (2019)	L	H	L	H	L	L	L	Poor
Vakili et al. (2019)	L	H	H	H	U	L	L	Poor
Samadi et al. (2020)	U	H	H	H	L	L	L	Poor
Chiu et al. (2021)	U	H	U	H	L	L	L	Poor
Hosseini et al. (2021)	L	H	L	H	H	L	L	Poor
Sanayei et al. (2021)	L	U	L	H	L	L	L	Fair
Tofighi et al. (2021)	L	H	L	H	H	L	L	Poor
Sanayei, Hajizadeh‐Sharafabad, et al. (2022)	L	H	L	L	L	L	L	Fair
Amiri Mandoulakani et al. (2023)	U	H	L	H	U	L	L	Poor
Sandgruber et al. (2023)	L	H	H	H	L	L	L	Poor
Delfan, Radkia, et al. (2024)	U	H	U	H	H	L	L	Poor
Delfan, Behzadi, et al. (2024)	L	H	U	H	L	L	L	Poor
Tofighi et al. (2024)	U	H	H	H	L	L	L	Poor

Abbreviations: H, high risk of bias; L, low risk of bias; U, unclear risk of bias.

### 3.4 Effects of *Chlorella* Supplementation on Anthropometric Measures

The result of our meta‐analysis indicated significant reductions in BF% (WMD = −0.72%; 95% CI: −1.11 to −0.34; *p* < 0.001; *I* ^2^ = 24.1%; *p* = 0.214) (Figure 2), BMI (WMD = −0.35 kg/m^2^; 95% CI: −0.55 to −0.14; *p* = 0.001; *I* ^2^ = 35.5%; *p* = 0.063), weight (WMD = −1.41 kg; 95% CI: −2.19 to −0.64; *p* < 0.001; *I* ^2^ = 25.7%; *p* = 0.128), and WHR (WMD = −0.01; 95% CI: −0.02 to −0.01; *p* < 0.001; *I* ^2^ = 0.0%; *p* = 0.766). Non‐significant effects were observed for HC (WMD = −0.21 cm; 95% CI: −1.80 to 1.39; *p* = 0.801; *I* ^2^ = 0.0%; *p* = 0.430) and WC (WMD = −0.30 cm; 95% CI: −2.06 to 1.46; *p* = 0.739; *I* ^2^ = 0.3%; *p* = 0.390).

The Egger's test indicated significant publication bias for WC (*p* = 0.049) and weight (*p* = 0.035). No publication bias was detected for BF% (*p* = 0.658), BMI (*p* = 0.421), HC (*p* = 0.827), or WHR (*p* = 0.228) based on Egger's test.

Sensitivity analysis revealed no influence of individual studies on any of the anthropometric parameters.

### 3.5 Effects of *Chlorella* Supplementation on Glycemic Profile

*Chlorella* supplementation demonstrated significant reductions in HOMA‐IR (WMD = −0.16 units; 95% CI: −0.29 to −0.04; *p* = 0.010; *I* ^2^ = 71.9%; *p* < 0.001) (Figure 3) and insulin levels (WMD = −1.00 μU/mL; 95% CI: −1.34 to −0.66; *p* < 0.001; *I* ^2^ = 27.5%; *p* = 0.199). In contrast, no significant effects were observed for FBG (WMD = −1.71 mg/dL; 95% CI: −5.21 to 1.79; *p* = 0.340; *I* ^2^ = 67.8%; *p* = 0.002).

No evidence of publication bias was detected for FBG (*p* = 0.932) and insulin levels (*p* = 0.083) based on Egger's test. However, publication bias was detected for HOMA‐IR (Egger's *p* = 0.010).

Sensitivity analysis revealed that the exclusion of the study by Hosseini et al. (2021) (WMD = −2.79 mg/dL, 95% CI: −5.00, −0.59) attenuated the overall effect on the FBG estimate. However, the results for HOMA‐IR and insulin levels remained robust in all sensitivity analyses.

### 3.6 Effects of *Chlorella* Supplementation on Lipid Profile

The pooled results demonstrated significant reductions in LDL‐C (WMD = −5.73 mg/dL; 95% CI: −8.77 to −2.70; *p* < 0.001; *I* ^2^ = 57.0%; *p* = 0.008) (Figure 4), TC (WMD = −6.62 mg/dL; 95% CI: −10.18 to −3.05; *p* < 0.001; *I* ^2^ = 46.6%; *p* = 0.038), and TG (WMD = −3.24 mg/dL; 95% CI: −6.25 to −0.23; *p* = 0.035; *I* ^2^ = 35.8%; *p* = 0.104). Conversely, HDL‐C showed a non‐significant increase (WMD = 1.54 mg/dL; 95% CI: −0.24 to 3.32; *p* = 0.090; *I* ^2^ = 50.0%; *p* = 0.020).

According to Egger's test, no evidence of publication bias was found for HDL‐C (*p* = 0.648), LDL‐C (*p* = 0.289), TC (*p* = 0.339), or TG (*p* = 0.968).

Sensitivity analyses indicated that the exclusion of the studies by Karbalamahdi et al. (2019) (WMD = 2.10 mg/dL, 95% CI: 0.38 to 3.81) and Sandgruber et al. (2023) (WMD = 1.82 mg/dL, 95% CI: 0.35 to 3.30) influenced the overall estimate for HDL‐C. Additionally, the overall significance of the pooled effect on TG was altered when any of the following studies were excluded: Delfan, Radkia, et al. (2024) (WMD = −3.27 mg/dL, 95% CI: −7.21 to 0.68), Delfan, Behzadi, et al. (2024) (WMD = −2.65 mg/dL, 95% CI: −6.05 to 0.75), Sandgruber et al. (2023) (WMD = −2.77 mg/dL, 95% CI: −5.69 to 0.15), and Chitsaz et al. (2016) (WMD = −2.92 mg/dL, 95% CI: −5.99 to 0.14). The findings for LDL‐C and TC were stable across all sensitivity analyses.

### 3.7 Effects of *Chlorella* Supplementation on Liver Function Tests

The findings from our analysis indicated significant reductions in ALT (WMD = −5.22 IU/L; 95% CI: −8.78 to −1.65; *p* = 0.004; *I* ^2^ = 80.2%; *p* < 0.001) (Figure 5) and AST (WMD = −3.45 IU/L; 95% CI: −5.00 to −1.90; *p* < 0.001; *I* ^2^ = 18.0%; *p* = 0.300), whereas ALP showed a non‐significant change (WMD = −15.28 IU/L; 95% CI: −45.57 to 15.01; *p* = 0.323; *I* ^2^ = 76.5%; *p* = 0.014).

Publication bias was detected for ALT based on Egger's test (*p* = 0.032) but not for AST (*p* = 0.335) and ALP (*p* = 0.242).

Sensitivity analysis revealed that excluding the study by Vakili et al. (2019) (WMD = −30.17 IU/L; 95% CI: −50.72 to −9.62) resulted in a significant reduction in ALP estimates. However, the significance of ALT and AST outcomes remained robust across all sensitivity analyses.

### 3.8 Effects of *Chlorella* Supplementation on Oxidative Stress Parameters

*Chlorella* supplementation resulted in significant increases in CAT activity (Hb WMD = 19.15 IU/g; 95% CI: 0.44 to 37.85; *p* = 0.045; *I* ^2^ = 99.5%; *p* < 0.001) (Figure 6) and SOD levels (WMD = 20.53 U/L; 95% CI: 15.03 to 26.02; *p* < 0.001; *I* ^2^ = 0.0%; *p* = 0.649). Non‐significant changes were observed for GPx activity (WMD = −3.30 U/L; 95% CI: −11.71 to 5.10; *p* = 0.441; *I* ^2^ = 98.8%; *p* < 0.001), MDA levels (WMD = −1.15 nmol/mL; 95% CI: −2.57 to 0.26; *p* = 0.111; *I* ^2^ = 99.9%; *p* < 0.001), and TAC (WMD = −0.09 mmol/L; 95% CI: −1.35 to 1.17; *p* = 0.886; *I* ^2^ = 99.7%; *p* < 0.001).

No publication bias was detected for any of the parameters based on the Egger's test including CAT (*p* = 0.382), GPx (*p* = 0.404), MDA (*p* = 0.293), SOD (*p* = 0.790), and TAC (*p* = 0.822).

Sensitivity analysis indicated that the exclusion of the studies conducted by Tofighi et al. (2021) (Hb WMD = 10.29 IU/g, 95% CI: −1.66, 22.23) and Panahi, Tavana, et al. (2012) (Hb WMD = 21.16 IU/g, 95% CI: −0.28, 42.60) influenced the pooled effect size for CAT, while the exclusion of the study by Tofighi et al. (2021) (WMD = −0.60 nmol/mL, 95% CI: −1.05, −0.16) affected MDA, and the same study (WMD = 0.38 mmol/L, 95% CI: 0.20, 0.56) also influenced TAC. In contrast, the results for GPx and SOD remained stable and were not influenced by any single study.

### 3.9 Effects of *Chlorella* Supplementation on Leptin

The supplementation of *Chlorella* led to a significant decrease in leptin levels (WMD = −0.66 ng/mL; 95% CI: −1.24 to −0.08; *p* = 0.025; *I* ^2^ = 0.0%; *p* = 0.416) (Figure 7).

No evidence of publication bias was detected based on Egger's test (*p* = 0.390).

Sensitivity analysis revealed that excluding the study by Delfan, Radkia, et al. (2024); Delfan, Behzadi, et al. (2024) (WMD = −0.28 ng/mL, 95% CI: −1.12, 0.56) had a significant impact on the overall result.

### 3.10 Subgroup Analysis

Subgroup analyses were conducted to assess the differential effects of *Chlorella* supplementation across country (Iran, other countries), health condition (healthy, chronic disease), sex (both, female, male), intervention age (< 40 years, ≥ 40 years), baseline BMI (overweight: 25 ≤ BMI < 30 kg/m^2^, individual with obesity: BMI ≥ 30 kg/m^2^), intervention duration (≤ 8 weeks, > 8 weeks), intervention type (tablet, capsule, other forms or not mentioned), intervention approach (*Chlorella*, *Chlorella* with exercise), *Chlorella* species (*Chlorella vulgaris, Chlorella pyrenoidosa*), control approach (placebo or usual care, exercise), *Chlorella* dosage (< 1500 or ≥ 1500 mg/day), and sample size (≤ 30 vs. > 30 participants), are summarized in Table 3.

**TABLE 3:** Description of the analysis and subgroup results of *Chlorella* supplementation in individuals with overweight and obesity.

	Studies N	Participant N	WMD (95% CI)	*p‐values*	Heterogeneity		
					*p* heterogeneity	*I* ^2^	*p* between sub‐groups
*Analysis and subgroup results of Chlorella supplementation on BF%*							
Overall effect	11	258	−0.72 (−1.11, −0.34)	< 0.001	0.214	24.1%	
Country							
Iran	10	214	−0.77 (−1.12, −0.43)	**< 0.001**	0.330	12.2%	0.089
Other countries	1	44	1.73 (−1.13, 4.59)	0.236	—	0.0%	
Health condition							
Healthy	9	218	−0.75 (−1.22, −0.27)	**0.002**	0.134	35.5%	0.751
Chronic diseases	2	40	−0.58 (−1.47, 0.31)	0.200	0.433	0.0%	
Sex							
Both sex	1	44	1.73 (−1.13, 4.59)	0.236	—	0.0%	0.211
Female	6	126	−0.62 (−0.97, −0.26)	**< 0.001**	0.730	0.0%	
Male	4	88	−0.98 (−1.97, 0.01)	0.052	0.192	36.7%	
Intervention age (year)							
< 40	6	134	−1.15 (−1.67, −0.63)	**< 0.001**	0.394	3.5%	0.049
≥ 40	5	124	−0.50 (−0.88, −0.12)	**0.010**	0.462	0.0%	
Baseline BMI							
Overweight (≥ 25 and < 30 kg/m^2^)	4	104	−0.44 (−0.88, 0.00)	0.051	0.362	6.2%	0.036
Obese (≥ 30 kg/m^2^)	7	154	−1.11 (−1.56, −0.66)	**< 0.001**	0.483	0.0%	
Intervention duration (weeks)							
≤ 8	6	126	−0.62 (−0.97, −0.26)	**< 0.001**	0.730	0.0%	0.998
> 8	5	132	−0.61 (−1.81, 0.58)	0.315	0.073	53.3%	
Intervention type							
Tablet	2	40	−0.53 (−0.96, −0.10)	**0.015**	0.437	0.0%	0.129
Capsule	6	134	−1.15 (−1.67, −0.63)	**< 0.001**	0.394	3.5%	
Other forms or not mentioned	3	84	−0.23 (−1.35, 0.90)	0.693	0.234	31.1%	
Intervention approach							
*Chlorella*	6	151	−0.82 (−1.47, −0.17)	**0.014**	0.086	48.2%	0.544
*Chlorella* with Exercise	5	107	−0.57 (−1.03, −0.12)	**0.014**	0.645	0.0%	
*Chlorella* species							
*Chlorella vulgaris*	10	214	−0.77 (−1.12, −0.43)	**< 0.001**	0.330	12.2%	0.089
*Chlorella pyrenoidosa*	1	44	1.73 (−1.13, 4.59)	0.236	—	0.0%	
Control approach							
Placebo or Usual care	6	151	−0.82 (−1.47, −0.17)	**0.014**	0.086	48.2%	0.544
Exercise	5	107	−0.57 (−1.03, −0.12)	**0.014**	0.645	0.0%	
*Chlorella* dosage (mg/day)							
< 1500	7	170	−0.58 (−0.94, −0.22)	**0.001**	0.500	0.0%	0.452
≥ 1500	4	88	−0.98 (−1.97, 0.01)	0.052	0.192	36.7%	
Sample size							
≤ 30	10	214	−0.77 (−1.12, −0.43)	**< 0.001**	0.330	12.2%	0.089
> 30	1	44	1.73 (−1.13, 4.59)	0.236	—	0.0%	
*Analysis and subgroup results of Chlorella supplementation on BMI*							
Overall effect	19	478	−0.35 (−0.55, −0.14)	**0.001**	0.063	35.5%	
Country							
Iran	18	434	−0.36 (−0.57, −0.15)	**< 0.001**	0.052	38.0%	0.440
Other countries	1	44	0.14 (−1.11, 1.39)	0.826	—	0.0%	
Health condition							
Healthy	15	322	−0.35 (−0.56, −0.13)	**0.002**	0.045	41.9%	0.945
Chronic diseases	4	156	−0.38 (−1.38, 0.62)	0.454	0.281	21.6%	
Sex							
Both sex	3	160	−0.46 (−1.72, 0.80)	0.473	0.136	49.8%	0.428
Female	8	150	−0.22 (−0.38, −0.06)	**0.007**	0.412	2.3%	
Male	8	168	−0.53 (−0.97, −0.08)	**0.020**	0.066	47.1%	
Intervention age (year)							
< 40	14	278	−0.36 (−0.58, −0.14)	**0.001**	0.035	44.9%	0.651
≥ 40	5	200	−0.18 (−0.91, 0.54)	0.618	0.381	4.5%	
Baseline BMI							
Overweight (≥ 25 and < 30 kg/m^2^)	7	243	−0.27 (−0.75, 0.22)	0.278	0.612	0.0%	0.717
Obese (≥ 30 kg/m^2^)	12	235	−0.37 (−0.61, −0.12)	**0.004**	0.015	53.0%	
Intervention duration (weeks)							
≤ 8	14	346	−0.24 (−0.39, −0.08)	**0.002**	0.580	0.0%	0.453
> 8	5	132	−0.48 (−1.08, 0.13)	0.123	0.007	71.4%	
Intervention type							
Tablet	4	93	−0.38 (−0.83, 0.08)	0.102	0.072	57.0%	0.568
Capsule	10	269	−0.44 (−0.84, −0.03)	**0.035**	0.103	38.3%	
Other forms or not mentioned	5	116	−0.21 (−0.43, 0.01)	0.061	0.424	0.0%	
Intervention approach							
*Chlorella*	10	299	−0.40 (−0.78, −0.01)	**0.042**	0.008	59.4%	0.826
*Chlorella* with exercise	9	179	−0.35 (−0.56, −0.14)	**0.001**	0.719	0.0%	
*Chlorella* species							
*Chlorella vulgaris*	18	434	−0.36 (−0.57, −0.15)	**< 0.001**	0.052	38.0%	0.440
*Chlorella pyrenoidosa*	1	44	0.14 (−1.11, 1.39)	0.826	—	0.0%	
Control approach							
Placebo or usual care	10	299	−0.40 (−0.78, −0.01)	**0.042**	0.008	59.4%	0.826
Exercise	9	179	−0.35 (−0.56, −0.14)	**0.001**	0.719	0.0%	
*Chlorella* dosage (mg/day)							
< 1500	14	315	−0.23 (−0.39, −0.08)	**0.003**	0.556	0.0%	0.387
≥ 1500	5	163	−0.50 (−1.10, 0.09)	0.096	0.009	70.2%	
Sample size							
≤ 30	16	318	−0.35 (−0.55, −0.14)	**0.001**	0.066	37.3%	0.862
> 30	3	160	−0.46 (−1.72, 0.80)	0.473	0.136	49.8%	
*Analysis and subgroup results of Chlorella supplementation on HC*							
Overall effect	3	121	−0.21 (−1.80, 1.39)	0.801	0.430	0.0%	
*Analysis and subgroup results of Chlorella supplementation on WC*							
Overall effect	4	162	−0.30 (−2.06, 1.46)	0.739	0.390	0.3%	
*Analysis and subgroup results of Chlorella supplementation on weight*							
Overall effect	23	564	−1.41 (−2.19, −0.64)	**< 0.001**	0.128	25.7%	
Country							
Iran	22	520	−1.49 (−2.27, −0.70)	**< 0.001**	0.133	25.7%	0.328
Other countries	1	44	0.43 (−3.33, 4.19)	0.823	—	0.0%	
Health condition							
Healthy	19	408	−1.42 (−2.29, −0.56)	**0.001**	0.069	34.6%	0.625
Chronic diseases	4	156	−0.80 (−3.15, 1.56)	0.507	0.713	0.0%	
Sex							
Both sex	3	160	−0.55 (−2.81, 1.71)	0.634	0.450	0.0%	0.168
Female	12	236	−0.48 (−1.62, 0.66)	0.407	1.000	0.0%	
Male	8	168	−2.13 (−3.48, −0.77)	**0.002**	0.011	61.4%	
Intervention age (year)							
< 40	16	324	−1.72 (−2.68, −0.75)	**< 0.001**	0.093	33.6%	0.122
≥ 40	7	240	−0.45 (−1.73, 0.82)	0.485	0.934	0.0%	
Baseline BMI							
Overweight (≥ 25 and < 30 kg/m^2^)	9	283	−0.56 (−1.75, 0.62)	0.352	0.975	0.0%	0.159
Obese (≥ 30 kg/m^2^)	14	281	−1.71 (−2.77, −0.64)	**0.002**	0.053	41.4%	
Intervention duration (weeks)							
≤ 8	18	432	−0.88 (−1.77, 0.02)	0.055	0.999	0.0%	0.316
> 8	5	132	−1.97 (−3.90, −0.03)	**0.047**	< 0.001	78.6%	
Intervention type							
Tablet	6	133	−0.86 (−2.20, 0.49)	0.211	0.898	0.0%	0.502
Capsule	12	315	−1.64 (−2.83, −0.45)	**0.007**	0.020	51.5%	
Other forms or not mentioned	5	116	−0.37 (−2.43, 1.70)	0.727	0.969	0.0%	
Intervention Approach							
*Chlorella*	12	342	−1.77 (−3.04, −0.49)	**0.007**	0.086	38.3%	0.242
*Chlorella* with exercise	11	222	−0.87 (−1.66, −0.09)	**0.030**	0.999	0.0%	
*Chlorella* Species							
*Chlorella vulgaris*	22	520	−1.49 (−2.27, −0.70)	**< 0.001**	0.133	25.7%	0.328
*Chlorella pyrenoidosa*	1	44	0.43 (−3.33, 4.19)	0.823	—	0.0%	
Control approach							
Placebo or usual care	12	342	−1.77 (−3.04, −0.49)	**0.007**	0.086	38.3%	0.242
Exercise	11	222	−0.87 (−1.66, −0.09)	**0.030**	0.999	0.0%	
*Chlorella* dosage (mg/day)							
< 1500	18	401	−0.86 (−1.76, 0.04)	0.062	0.999	0.0%	0.281
≥ 1500	5	163	−2.01 (−3.91, −0.12)	**0.037**	0.001	78.2%	
Sample size							
≤ 30	20	404	−1.48 (−2.32, −0.65)	**< 0.001**	0.109	29.1%	0.448
> 30	3	160	−0.55 (−2.81, 1.71)	0.634	0.450	0.0%	
*Analysis and subgroup results of Chlorella supplementation on WHR*							
Overall effect	6	126	−0.01 (−0.02, −0.01)	**< 0.001**	0.766	0.0%	
*Analysis and subgroup results of Chlorella supplementation on FBG*							
Overall effect	9	251	−1.71 (−5.21, 1.79)	0.340	0.002	67.8%	
Country							
Iran	8	215	−2.05 (−6.35, 2.24)	0.349	0.002	69.5%	0.452
Other countries	1	36	0.00 (−3.18, 3.18)	1.000	—	0.0%	
Health condition							
Healthy	8	176	−2.80 (−5.00, −0.59)	**0.013**	0.261	21.2%	< 0.001
Chronic diseases	1	75	13.92 (5.98, 21.86)	**< 0.001**	—	0.0%	
Sex							
Both sex	2	111	6.46 (−7.14, 20.07)	0.352	0.001	90.2%	0.049
Female	2	32	−0.11 (−4.43, 4.21)	0.961	0.821	0.0%	
Male	5	108	−5.08 (−7.54, −2.63)	**< 0.001**	0.898	0.0%	
Intervention age (year)							
< 40	8	176	−2.80 (−5.00, −0.59)	**0.013**	0.261	21.2%	< 0.001
≥ 40	1	75	13.92 (5.98, 21.86)	**< 0.001**	—	0.0%	
Baseline BMI							
Overweight (≥ 25 and < 30 kg/m^2^)	2	95	4.37 (−13.56, 22.31)	0.633	< 0.001	94.4%	0.488
Obese (≥ 30 kg/m^2^)	7	156	−2.03 (−4.55, 0.49)	0.114	0.339	11.9%	
Intervention duration (weeks)							
≤ 8	5	163	0.97 (−3.63, 5.56)	0.680	< 0.001	79.2%	0.020
> 8	4	88	−6.63 (−11.07, −2.19)	**0.003**	0.940	0.0%	
Intervention type							
Tablet	3	52	−2.59 (−5.68, 0.50)	0.101	0.267	24.2%	0.503
Capsule	5	163	−2.52 (−11.23, 6.19)	0.571	< 0.001	80.0%	
Other forms or not mentioned	1	36	0.00 (−3.18, 3.18)	1.000	—	0.0%	
Intervention approach							
*Chlorella*	5	171	−0.22 (−6.49, 6.06)	0.946	0.002	77.0%	0.295
*Chlorella* with exercise	4	80	−3.81 (−6.22, −1.40)	**0.002**	0.498	0.0%	
*Chlorella* species							
*Chlorella vulgaris*	8	215	−2.05 (−6.35, 2.24)	0.349	0.002	69.5%	0.452
*Chlorella pyrenoidosa*	1	36	0.00 (−3.18, 3.18)	1.000	—	0.0%	
Control approach							
Placebo or usual care	5	171	−0.22 (−6.49, 6.06)	0.946	0.002	77.0%	0.295
Exercise	4	80	−3.81 (−6.22, −1.40)	**0.002**	0.498	0.0%	
*Chlorella* dosage (mg/day)							
< 1500	3	52	−2.59 (−5.68, 0.50)	0.101	0.267	24.2%	0.834
≥ 1500	6	199	−1.87 (−7.86, 4.13)	0.542	0.001	75.6%	
Sample size							
≤ 30	7	140	−3.87 (−6.00, −1.73)	**< 0.001**	0.547	0.0%	0.141
> 30	2	111	6.46 (−7.14, 20.07)	0.352	0.001	90.2%	
*Analysis and subgroup results of Chlorella supplementation on HOMA‐IR*							
Overall effect	9	235	−0.16 (−0.29, −0.04)	**0.010**	< 0.001	71.9%	
Country							
Iran	9	235	−0.16 (−0.29, −0.04)	**0.010**	< 0.001	71.9%	
Other countries	0	0	—	—	—	—	
Health condition							
Healthy	8	160	−0.17 (−0.30, −0.04)	**0.009**	< 0.001	75.4%	0.498
Chronic diseases	1	75	0.05 (−0.58, 0.67)	0.883	—	—	
Sex							
Both sex	1	75	0.05 (−0.58, 0.67)	0.883	—	—	0.085
Female	2	32	−0.02 (−0.13, 0.09)	0.687	0.282	13.7%	
Male	6	128	−0.31 (−0.54, −0.08)	**0.010**	< 0.001	81.6%	
Intervention age (year)							
< 40	8	160	−0.17 (−0.30, 0.04)	**0.009**	< 0.001	75.4%	0.498
≥ 40	1	75	0.05 (−0.58, 0.67)	0.883	—	—	
Baseline BM**I**							
Overweight (≥ 25 and < 30 kg/m^2^)	3	115	−0.11 (−0.36, 0.15)	0.422	0.067	62.9%	0.461
Obese (≥ 30 kg/m^2^)	6	120	−0.23 (−0.41, −0.04)	**0.017**	0.004	71.0%	
Intervention duration (weeks)							
≤ 8	5	147	−0.03 (−0.11, 0.05)	0.444	0.153	40.3%	0.004
> 8	4	88	−0.39 (−0.61, −0.16)	**0.001**	0.175	39.4%	
Intervention Type							
Tablet	4	72	−0.04 (−0.13, 0.05)	0.410	0.083	55.1%	0.010
Capsule	5	163	−0.35 (−0.57, −0.13)	**0.002**	0.171	37.5%	
Other forms or not mentioned	0	0	—	—	—	—	
Intervention approach							
*Chlorella*	5	155	−0.13 (−0.32, 0.06)	0.174	0.005	73.2%	0.589
*Chlorella* with exercise	4	80	−0.19 (−0.32, −0.06)	**0.004**	0.265	24.4%	
*Chlorella* species							
*Chlorella vulgaris*	9	235	−0.16 (−0.29, −0.04)	**0.010**	< 0.001	71.9%	
*Chlorella pyrenoidosa*	0	0	—	—	—	—	
Control approach							
Placebo or usual care	5	155	−0.13 (−0.32, 0.06)	0.174	0.005	73.2%	0.589
Exercise	4	80	−0.19 (−0.32, −0.06)	**0.004**	0.265	24.4%	
*Chlorella* dosage (mg/day)							
< 1500	4	72	−0.04 (−0.13, 0.05)	0.410	0.083	55.1%	0.010
≥ 1500	5	163	−0.35 (−0.57, −0.13)	**0.002**	0.171	37.5%	
Sample size							
≤ 30	8	160	−0.17 (−0.30, −0.04)	**0.009**	< 0.001	75.4%	0.498
> 30	1	75	−0.05 (−0.58, 0.67)	0.883	—	—	
*Analysis and subgroup results of Chlorella supplementation on insulin*							
Overall effect	9	251	−1.00 (−1.34, −0.66)	**< 0.001**	0.199	27.5%	
Country							
Iran	8	215	−1.09 (−1.36, −0.81)	**< 0.001**	0.401	3.7%	0.053
Other countries	1	36	0.60 (−1.08, 2.28)	0.485	—	0.0%	
Health condition							
Healthy	8	176	−1.00 (−1.37, −0.63)	**< 0.001**	0.144	35.7%	0.738
Chronic diseases	1	75	−0.68 (−2.49, 1.12)	0.458	—	0.0%	
Sex							
Both sex	2	111	0.00 (−1.25, 1.26)	0.998	0.309	3.6%	0.055
Female	2	32	−0.14 (−1.26, 0.99)	0.813	0.633	0.0%	
Male	5	108	−1.16 (−1.44, −0.88)	**< 0.001**	0.427	0.0%	
Intervention age (year)							
< 40	8	176	−1.00 (−1.37, −0.63)	**< 0.001**	0.144	35.7%	0.738
≥ 40	1	75	−0.68 (−2.49, 1.12)	0.458	—	0.0%	
Baseline BMI							
Overweight (≥ 25 and < 30 kg/m^2^)	2	95	−0.84 (−1.80, 0.12)	0.086	0.842	0.0%	0.777
Obese (≥ 30 kg/m^2^)	7	156	−0.99 (−1.40, −0.58)	**< 0.001**	0.095	44.5%	
Intervention duration (weeks)							
≤ 8	5	163	−0.36 (−1.03, 0.31)	0.289	0.622	0.0%	0.030
> 8	4	88	−1.18 (−1.50, −0.86)	**< 0.001**	0.303	17.5%	
Intervention type							
Tablet	3	52	−0.52 (−1.31, 0.28)	0.206	0.574	0.0%	0.049
Capsule	5	163	−1.16 (−1.45, −0.88)	**< 0.001**	0.418	0.0%	
Other forms or not mentioned	1	36	0.60 (−1.08, 2.28)	0.485	—	—	
Intervention approach							
*Chlorella*	5	171	−0.86 (−1.60, −0.11)	**0.024**	0.065	54.8%	0.781
*Chlorella* with exercise	4	80	−0.97 (−1.31, −0.64)	**< 0.001**	0.641	0.0%	
*Chlorella* species							
*Chlorella vulgaris*	8	215	−1.09 (−1.36, −0.81)	**< 0.001**	0.401	3.7%	0.053
*Chlorella pyrenoidosa*	1	36	0.60 (−1.08, 2.28)	0.485	—	0.0%	
Control approach							
Placebo or usual care	5	171	−0.86 (−1.60, −0.11)	**0.024**	0.065	54.8%	0.781
Exercise	4	80	−0.97 (−1.31, −0.64)	**< 0.001**	0.641	0.0%	
*Chlorella* dosage (mg/day)							
< 1500	3	52	−0.52 (−1.31, 0.28)	0.206	0.574	0.0%	0.202
≥ 1500	6	199	−1.09 (−1.48, −0.70)	**< 0.001**	0.156	37.5%	
Sample size							
≤ 30	7	140	−1.09 (−1.40, −0.78)	**< 0.001**	0.314	15.2%	0.097
> 30	2	111	0.00 (−1.25, 1.26)	0.998	0.309	3.6%	
*Analysis and subgroup results of Chlorella supplementation on HDL‐C*							
Overall effect	13	378	1.54 (−0.24, 3.32)	0.090	0.020	50.0%	
Country							
Iran	11	298	1.64 (0.13, 3.16)	**0.033**	0.161	30.0%	0.557
Other countries	2	80	−3.13 (−19.00, 12.74)	0.699	0.002	89.4%	
Health condition							
Healthy	11	262	1.74 (−0.36, 3.85)	0.105	0.009	57.7%	0.488
Chronic diseases	2	116	0.46 (−2.50, 3.42)	0.761	0.806	0.0%	
Sex							
Both sex	4	196	−0.88 (−6.32, 4.56)	0.751	0.023	68.6%	0.028
Female	5	94	0.47 (−1.40, 2.33)	0.623	0.282	20.8%	
Male	4	88	4.18 (1.91, 6.45)	**< 0.001**	0.952	0.0%	
Intervention age (year)							
< 40	10	218	1.49 (−0.73, 3.71)	0.189	0.008	59.4%	0.958
≥ 40	3	160	1.40 (−1.21, 4.00)	0.293	0.414	0.0%	
Baseline BMI							
Overweight (≥ 25 and < 30 kg/m^2^)	4	183	1.74 (−0.63, 4.12)	0.150	0.536	0.0%	0.794
Obese (≥ 30 kg/m^2^)	9	195	1.30 (−1.09, 3.68)	0.287	0.006	62.8%	
Intervention duration (weeks)							
≤ 8	8	246	0.14 (−1.85, 2.14)	0.889	0.102	41.4%	0.005
> 8	5	132	4.24 (2.14, 6.34)	**< 0.001**	0.985	0.0%	
Intervention type							
Tablet	1	41	−0.65 (−9.99, 8.69)	0.892	—	0.0%	0.159
Capsule	7	209	3.00 (1.35, 4.65)	**< 0.001**	0.715	0.0%	
Other forms or not mentioned	5	128	−0.31 (−3.46, 2.85)	0.849	0.023	64.7%	
Intervention approach							
*Chlorella*	8	279	1.85 (−0.69, 4.39)	0.152	0.030	54.9%	0.309
*Chlorella* with exercise	5	99	0.22 (−1.62, 2.07)	0.811	0.348	10.2%	
*Chlorella* species							
*Chlorella vulgaris*	11	298	1.64 (0.13, 3.16)	**0.033**	0.161	30.0%	0.557
*Chlorella pyrenoidosa*	2	80	−3.13 (−19.00, 12.74)	0.699	0.002	89.4%	
Control approach							
Placebo or usual care	8	279	1.85 (−0.69, 4.39)	0.152	0.030	54.9%	0.309
Exercise	5	99	0.22 (−1.62, 2.07)	0.811	0.348	10.2%	
*Chlorella* dosage (mg/day)							
< 1500	7	179	0.85 (−0.94, 2.64)	0.350	0.277	20.1%	0.708
≥ 1500	6	199	1.58 (−1.78, 4.94)	0.357	0.017	63.9%	
Sample size							
≤ 30	9	182	2.01 (0.16, 3.86)	**0.033**	0.084	42.5%	0.325
> 30	4	196	−0.88 (−6.32, 4.56)	0.751	0.023	68.6%	
*Analysis and subgroup results of Chlorella supplementation on LDL‐C*							
Overall effect	12	362	−5.73 (−8.77, −2.70)	**< 0.001**	0.008	57.0%	
Country							
Iran	10	282	−4.34 (−7.07, −1.62)	**0.002**	0.066	43.9%	0.004
Other countries	2	80	−14.84 (−21.46, −8.23)	**< 0.001**	0.441	0.0%	
Health condition							
Healthy	10	246	−6.39 (−8.91, −3.87)	**< 0.001**	0.141	33.3%	0.448
Chronic diseases	2	116	−0.69 (−15.21, 13.82)	0.926	0.027	79.6%	
Sex							
Both sex	4	196	−8.35 (−20.45, 3.75)	0.176	< 0.001	85.2%	0.680
Female	4	78	−7.13 (−13.08, −1.19)	**0.019**	0.287	20.5%	
Male	4	88	−4.85 (−6.94, −2.76)	**< 0.001**	0.808	0.0%	
Intervention age (year)							
< 40	9	202	−5.87 (−8.10, −3.64)	**< 0.001**	0.266	19.9%	0.929
≥ 40	3	160	−6.57 (−21.94, 8.80)	0.402	0.002	83.9%	
Baseline BMI							
Overweight (≥ 25 and < 30 kg/m^2^)	4	183	−8.33 (−20.82, 4.15)	0.191	< 0.001	82.7%	0.656
Obese (≥ 30 kg/m^2^)	8	179	−5.46 (−7.49, −3.43)	**< 0.001**	0.372	7.6%	
Intervention duration (weeks)							
≤ 8	7	230	−6.27 (−12.86, 0.32)	0.062	0.002	71.0%	0.763
> 8	5	132	−5.19 (−7.55, −2.84)	**< 0.001**	0.298	18.3%	
Intervention type							
Tablet	1	41	−8.90 (−20.33, 2.53)	0.127	—	0.0%	0.420
Capsule	7	209	−4.30 (−7.59, −1.01)	**0.010**	0.023	59.0%	
Other forms or not mentioned	4	112	−9.23 (−16.93, −1.53)	**0.019**	0.072	57.2%	
Intervention approach							
*Chlorella*	8	279	−6.53 (−11.22, −1.83)	**0.006**	0.001	71.0%	0.441
*Chlorella* with exercise	4	83	−4.34 (−7.33, −1.35)	**0.004**	0.791	0.0%	
*Chlorella* species							
*Chlorella vulgaris*	10	282	−4.34 (−7.07, −1.62)	**0.002**	0.066	43.9%	0.004
*Chlorella pyrenoidosa*	2	80	−14.84 (−21.46, −8.23)	**< 0.001**	0.441	0.0%	
Control approach							
Placebo or usual care	8	279	−6.53 (−11.22, −1.83)	**0.006**	0.001	71.0%	0.441
Exercise	4	83	−4.34 (−7.33, −1.35)	**0.004**	0.791	0.0%	
*Chlorella* dosage (mg/day)							
< 1500	6	163	−8.79 (−13.99, −3.59)	**< 0.001**	0.271	21.7%	0.171
≥ 1500	6	199	−4.36 (−8.00, −0.72)	**0.019**	0.005	69.9%	
Sample size							
≤ 30	8	166	−5.15 (−7.09, −3.21)	**< 0.001**	0.621	0.0%	0.609
> 30	4	196	−8.35 (−20.45, 3.75)	0.176	< 0.001	85.2%	
*Analysis and subgroup results of Chlorella supplementation on TC*							
Overall effect	12	362	−6.62 (−10.18, −3.05)	**< 0.001**	0.038	46.6%	
Country							
Iran	10	282	−5.70 (−9.37, −2.02)	**0.002**	0.051	46.5%	0.067
Other countries	2	80	−14.91 (−24.05, −5.78)	**0.001**	0.324	0.0%	
Health condition							
Healthy	10	246	−8.19 (−11.08, −5.31)	**< 0.001**	0.396	4.8%	0.446
Chronic diseases	2	116	−1.80 (−18.00, 14.41)	0.828	0.002	89.3%	
Sex							
Both sex	4	196	−7.23 (−17.74, 3.29)	0.178	0.003	79.0%	0.852
Female	4	78	−5.62 (−11.45, 0.21)	0.059	0.704	0.0%	
Male	4	88	−7.69 (−11.89, −3.48)	**< 0.001**	0.235	29.5%	
Intervention age (year)							
< 40	9	202	−7.98 (−11.19, −4.78)	**< 0.001**	0.306	15.3%	0.497
≥ 40	3	160	−3.75 (−15.53, 8.02)	0.532	0.008	79.4%	
Baseline BMI							
Overweight (≥ 25 and < 30 kg/m^2^)	4	183	−5.19 (−13.93, 3.56)	0.245	0.015	71.2%	0.610
Obese (≥ 30 kg/m^2^)	8	179	−7.65 (−11.28, −4.02)	**< 0.001**	0.228	25.2%	
Intervention duration (weeks)							
≤ 8	7	230	−6.11 (−12.26, 0.04)	0.052	0.016	61.6%	0.564
> 8	5	132	−8.18 (−11.62, −4.75)	**< 0.001**	0.371	6.2%	
Intervention type							
Tablet	1	41	−9.76 (−15.78, −3.74)	**0.001**	—	0.0%	0.531
Capsule	7	209	−5.33 (−10.31, −0.36)	**0.036**	0.023	59.1%	
Other forms or not mentioned	4	112	−7.98 (−15.98, 0.02)	0.051	0.165	41.2%	
Intervention approach							
*Chlorella*	8	279	−7.76 (−12.25, −3.26)	**< 0.001**	0.016	59.2%	0.195
*Chlorella* with exercise	4	83	−2.92 (−8.70, 2.87)	0.323	0.875	0.0%	
*Chlorella* Species							
*Chlorella vulgaris*	10	282	−5.70 (−9.37, −2.02)	**0.002**	0.051	46.5%	0.067
*Chlorella pyrenoidosa*	2	80	−14.91 (−24.05, −5.78)	**0.001**	0.324	0.0%	
control approach							
Placebo or usual care	8	279	−7.76 (−12.25, −3.26)	**< 0.001**	0.016	59.2%	0.195
Exercise	4	83	−2.92 (−8.70, 2.87)	0.323	0.875	0.0%	
*Chlorella* dosage (mg/day)							
< 1500	6	163	−7.73 (−11.76, −3.70)	**< 0.001**	0.796	0.0%	0.634
≥ 1500	6	199	−5.93 (−12.11, 0.25)	0.060	0.003	72.5%	
Sample size							
≤ 30	8	166	−7.62 (−10.52, −4.73)	**< 0.001**	0.509	0.0%	0.943
> 30	4	196	−7.23 (−17.74, 3.29)	0.178	0.003	79.0%	
*Analysis and subgroup results of Chlorella supplementation on TG*							
Overall effect	12	362	−3.24 (−6.25, −0.23)	**0.035**	0.104	35.8%	
Country							
Iran	10	282	−2.88 (−5.86, 0.10)	0.058	0.120	36.1%	0.811
Other countries	2	80	−5.53 (−27.01, 15.96)	0.614	0.185	43.1%	
Health condition							
Healthy	10	246	−3.22 (−5.71, −0.72)	**0.011**	0.252	20.8%	0.668
Chronic diseases	2	116	3.05 (−25.47, 31.56)	0.834	0.017	82.3%	
Sex							
Both sex	4	196	−1.22 (−16.13, 13.70)	0.873	0.037	64.6%	0.955
Female	4	78	−4.40 (−18.78, 9.97)	0.548	0.189	37.1%	
Male	4	88	−2.75 (−4.85, −0.65)	**0.010**	0.335	11.7%	
Intervention age (year)							
< 40	9	202	−3.32 (−5.88, −0.77)	**0.011**	0.225	24.6%	0.460
≥ 40	3	160	4.77 (−16.53, 26.08)	0.661	0.046	67.6%	
Baseline BMI							
Overweight (≥ 25 and < 30 kg/m^2^)	4	183	−1.18 (−18.55, 16.20)	0.894	0.037	64.6%	0.834
Obese (≥ 30 kg/m^2^)	8	179	−3.05 (−5.41, −0.68)	**0.011**	0.281	18.8%	
Intervention duration (weeks)							
≤ 8	7	230	−4.01 (−14.09, 6.06)	0.435	0.057	50.9%	0.798
> 8	5	132	−2.67 (−4.68, −0.67)	**0.009**	0.391	2.8%	
Intervention type							
Tablet	1	41	−10.65 (−24.45, 3.15)	0.130	—	0.0%	0.539
Capsule	7	209	−2.66 (−5.58, 0.26)	0.075	0.127	39.6%	
Other forms or not mentioned	4	112	−2.52 (−17.39, 12.34)	0.739	0.144	44.6%	
Intervention approach							
*Chlorella*	8	279	−3.78 (−8.33, 0.78)	0.104	0.082	44.6%	0.796
*Chlorella* with Exercise	4	83	−2.90 (−7.69, 1.89)	0.235	0.222	31.8%	
*Chlorella* species							
*Chlorella vulgaris*	10	282	−2.88 (−5.86, 0.10)	0.058	0.120	36.1%	0.811
*Chlorella pyrenoidosa*	2	80	−5.53 (−27.01, 15.96)	0.614	0.185	43.1%	
Control approach							
Placebo or usual care	8	279	−3.78 (−8.33, 0.78)	0.104	0.082	44.6%	0.796
Exercise	4	83	−2.90 (−7.69, 1.89)	0.235	0.222	31.8%	
*Chlorella* dosage (mg/day)							
< 1500	6	163	−5.30 (−15.19, 4.58)	0.293	0.277	20.8%	0.649
≥ 1500	6	199	−2.90 (−6.03, 0.23)	0.070	0.069	51.1%	
Sample size							
≤ 30	8	166	−2.89 (−5.22, −0.56)	**0.015**	0.304	16.0%	0.828
> 30	4	196	−1.22 (−16.13, 13.70)	0.873	0.037	64.6%	
*Analysis and subgroup results of Chlorella supplementation on ALP*							
Overall effect	3	81	−15.28 (−45.57, 15.01)	0.323	0.014	76.5%	
*Analysis and subgroup results of Chlorella supplementation on ALT*							
Overall effect	5	161	−5.22 (−8.78, −1.65)	**0.004**	< 0.001	80.2%	
*Analysis and subgroup results of Chlorella supplementation on AST*							
Overall effect	5	161	−3.45 (−5.00, −1.90)	**< 0.001**	0.300	18.0%	
*Analysis and subgroup results of Chlorella supplementation on CAT*							
Overall effect	4	141	19.15 (0.44, 37.85)	**0.045**	< 0.001	99.5%	
*Analysis and subgroup results of Chlorella supplementation on GPx*							
Overall effect	6	181	−3.30 (−11.71, 5.10)	0.441	< 0.001	98.8%	
*Analysis and subgroup results of Chlorella supplementation on MDA*							
Overall effect	9	221	−1.15 (−2.57, 0.26)	0.111	< 0.001	99.9%	
Country							
Iran	9	221	−1.15 (−2.57, 0.26)	0.111	< 0.001	99.9%	—
Other countries	0	—	—	—	—	—	
Health condition							
Healthy	8	164	−1.13 (−2.60, 0.34)	0.131	< 0.001	99.9%	0.873
Chronic diseases	1	57	−1.43 (−4.77, 1.91)	0.401	—	0.0%	
Sex							
Both sex	1	57	−1.43 (−4.77, 1.91)	0.401	—	0.0%	0.210
Female	2	40	−0.05 (−0.37, 0.26)	0.743	0.010	84.7%	
Male	6	124	−1.49 (−3.24, 0.26)	0.095	< 0.001	99.9%	
Intervention age (year)							
< 40	6	124	−1.49 (−3.24, 0.26)	0.095	< 0.001	99.9%	0.115
≥ 40	3	97	−0.06 (−0.37, 0.24)	0.684	0.027	72.4%	
Baseline BMI							
Overweight (≥ 25 and < 30 kg/m^2^)	3	97	−0.06 (−0.37, 0.24)	0.684	0.027	72.4%	0.115
Obese (≥ 30 kg/m^2^)	6	124	−1.49 (−3.24, 0.26)	0.095	< 0.001	99.9%	
Intervention duration (weeks)							
≤ 8	5	137	−1.51 (−3.83, 0.82)	0.205	< 0.001	99.9%	0.538
> 8	4	84	−0.75 (−1.39, −0.11)	**0.022**	< 0.001	97.8%	
Intervention type							
Tablet	3	97	−0.06 (−0.37, 0.24)	0.684	0.027	72.4%	0.115
Capsule	6	124	−1.49 (−3.24, 0.26)	0.095	< 0.001	99.9%	
Other forms or not mentioned	0	—	—	—	—	—	
Intervention approach							
*Chlorella*	5	139	−0.84 (−1.40, −0.29)	**0.003**	< 0.001	97.2%	0.683
*Chlorella* with exercise	4	82	−1.44 (−4.26, 1.38)	0.317	< 0.001	99.9%	
*Chlorella* species							
*Chlorella vulgaris*	9	221	−1.15 (−2.57, 0.26)	0.111	< 0.001	99.9%	—
*Chlorella pyrenoidosa*	0	—	—	—	—	—	
Control approach							
Placebo or usual care	5	139	−0.84 (−1.40, −0.29)	**0.003**	< 0.001	97.2%	0.683
Exercise	4	82	−1.44 (−4.26, 1.38)	0.317	< 0.001	99.9%	
*Chlorella* dosage (mg/day)							
< 1500	6	120	−1.11 (−3.04, 0.81)	0.257	< 0.001	99.9%	0.932
≥ 1500	3	101	−1.20 (−2.07, −0.34)	**0.006**	< 0.001	96.7%	
Sample size							
≤ 30	8	164	−1.13 (−2.60, 0.34)	0.131	< 0.001	99.9%	0.873
> 30	1	57	−1.43 (−4.77, 1.91)	0.401	—	0.0%	
*Analysis and subgroup results of Chlorella supplementation on SOD*							
Overall effect	4	84	20.53 (15.03, 26.02)	**< 0.001**	0.649	0.0%	
*Analysis and subgroup results of Chlorella supplementation on TAC*							
Overall effect	6	124	−0.09 (−1.35, 1.17)	0.886	< 0.001	99.7%	
*Analysis and subgroup results of Chlorella supplementation on leptin*							
Overall effect	3	60	−0.66 (−1.24, −0.08)	**0.025**	0.416	0.0%	

*Note:* The bold values indicate statistically significant differences with p‐value < 0.05.

Abbreviations: ALP, alkaline phosphatase; ALT, alanine aminotransferase; AST, aspartate aminotransferase; BF%, body fat percentage; BMI, body mass index; CAT, catalase; CI, confidence interval; FBG, fasting blood glucose; GPx, glutathione peroxidase; HC, hid circumference; HDL‐C, high‐density lipoprotein cholesterol; HOMA‐IR, homeostatic model assessment of insulin resistance; LDL‐C, low‐density lipoprotein cholesterol; MDA, malondialdehyde; N, number; SOD, superoxide dismutase; TAC, total antioxidant capacity; TC, total cholesterol; TG, triglycerides; WC, waist circumference; WHR, waist‐to‐hip ratio; WMD, weighted mean difference.

When stratified by country, *Chlorella* supplementation resulted in significant changes in BF%, BMI, weight, HOMA‐IR, insulin, and HDL‐C levels in studies conducted in Iran. In contrast, non‐Iranian studies showed significant reductions in FBG, TG, and MDA levels. Notably, effects on LDL‐C were significant in both subgroups.

In healthy populations, *Chlorella* supplementation was associated with significant reductions in BF%, BMI, weight, HOMA‐IR, insulin, LDL‐C, TC, and TG. Conversely, among participants with chronic diseases, significant reductions were observed only in HDL‐C and MDA. Significant effects on LDL‐C and TC were evident in both groups, suggesting that lipid‐lowering effects may be independent of baseline health status.

Furthermore, significant effects on weight, FBG, HOMA‐IR, insulin, HDL‐C, TC, and TG were observed in males. In contrast, females exhibited a significant reduction in BF%. Notably, BMI and LDL‐C showed significant changes in both male and female subgroups, while no significant changes were detected in studies involving participants of both sexes.

Subgroup analyses stratified by age revealed that participants aged < 40 years experienced significant reductions in BMI, weight, HOMA‐IR, insulin, LDL‐C, TC, and TG. Significant impacts in BF% and FBG were observed in both age groups (< 40 and ≥ 40 years). No significant changes were found in HDL‐C and MDA levels in either subgroup.

*Chlorella* supplementation demonstrated varying levels of effectiveness across baseline BMI.

Categories. In individuals with obesity (BMI ≥ 30 kg/m^2^), it led to significant reductions in BF%, BMI, weight, HOMA‐IR, insulin, LDL‐C, TC, and TG. However, FBG, HDL‐C, and MDA levels remained unaffected in both BMI subgroups.

Moreover, subgroup analysis based on intervention duration showed that shorter interventions (≤ 8 weeks) led to significant reductions in BF% and BMI. In contrast, longer durations (> 8 weeks) demonstrated broader efficacy, with significant changes observed in weight, FBG, HOMA‐IR, insulin, HDL‐C, LDL‐C, TC, TG, and MDA.

*Chlorella* supplementation in capsule form was associated with significant reductions in BMI, weight, HOMA‐IR, insulin, and HDL‐C. Both tablet and capsule formulations led to significant effects on BF% and TC. However, no significant effects on FBG, TG, or MDA were observed with any formulation.

Subgroup analyses comparing *Chlorella* monotherapy versus combined interventions with exercise indicated that *Chlorella* alone significantly reduced TC and MDA, while its combination with exercise led to significant impacts on FBG and HOMA‐IR levels. Significant changes in BF%, BMI, weight, insulin, and LDL‐C were observed in both monotherapy and combination subgroups. No significant changes in HDL‐C and TG were found in either group.

Subgroup analyses stratified by *Chlorella* species revealed that *Chlorella vulgaris* supplementation significantly reduced BF%, BMI, weight, HOMA‐IR, insulin, and HDL‐C. Both species shared lipid‐lowering effects by significant reductions in LDL‐C and TC. No significant effects were observed on FBG, TG, or MDA in either subgroup.

Variation in control approaches highlighted that both placebo or usual care and exercise comparators were associated with significant reductions in BF%, BMI, weight, insulin, LDL‐C, TC, and MDA. Significant changes in FBG and HIMA‐IR emerged only in studies using exercise as the comparator. HDL‐C and TG remained unchanged regardless of the control approach.

Additionally, when stratified by *Chlorella* dosage, lower doses (< 1500 mg/day) led to significant changes in BF%, BMI, insulin, and TC, while higher doses (≥ 1500 mg/day) showed greater efficacy in reducing weight, HOMA‐IR, and MDA. Both dosage groups were effective in lowering LDL‐C levels. No significant effects on FBG, HDL‐C, or TG were observed in either subgroup.

Studies with a smaller sample size (≤ 30 participants) demonstrated significant reductions in BF%, BMI, weight, FBG, HOMA‐IR, insulin, HDL‐C, LDL‐C, TC, and TG. However, MDA levels did not significantly change in either small or large sample size subgroups.

### 3.11 Meta‐Regression and Non‐Linear Dose–Response Analysis

In the non‐linear dose–response analysis, a significant association was found between *Chlorella* dosage and insulin level reduction (*p* = 0.026), with an approximate optimal dose estimated at 1800 mg/day. For HDL‐C, a significant inverse relationship with *Chlorella* dosage was observed (*p* < 0.001), with an estimated optimal dose of approximately 900 mg/day. A significant negative association was also identified between *Chlorella* dosage and LDL‐C levels (*p* = 0.005), with an optimal dose estimated at 1800 mg/day. A significant association was observed between intervention duration and weight (*p* = 0.031), with the most favorable effect seen around 8 weeks. Additionally, a significant association was noted between intervention duration and insulin (*p* = 0.079), with the optimal duration estimated at 6 weeks. Moreover, HDL‐C levels showed a significant increase with prolonged intervention periods (*p* = 0.014), particularly evident after 8 weeks (Figures S2 and S3).

The meta‐regression analysis identified a significant inverse association between *Chlorella* dosage and HDL‐C levels (*p* < 0.001), suggesting that higher dosages may reduce HDL‐C concentrations. Furthermore, increased dosages were associated with greater reductions in weight (*p* = 0.008). A significant relationship was also found between intervention duration and insulin levels (*p* = 0.007), indicating that prolonged supplementation may lead to notable decreases in insulin concentrations. Additionally, a significant association was found between intervention duration and HDL‐C levels (*p* = 0.012), suggesting that longer intervention periods may lead to an increase in HDL‐C levels (Figures S4 and S5).

### 3.12 GRADE Assessment

The GRADE assessment for outcomes related to *Chlorella* supplementation is presented in Table 4. Overall, the quality of evidence was rated as very low for most anthropometric indices (BF%, HC, WC, weight, WHR), glycemic markers (FBG, HOMA‐IR, insulin), lipid parameters (HDL‐C, LDL‐C, TC, TG), liver function enzymes (ALP, ALT, AST), oxidative stress markers (CAT, GPx, MDA, SOD, TAC), and leptin levels. In contrast, the quality of evidence for BMI was rated as low.

**TABLE 4:** Grade profile of *Chlorella* supplementation in individuals with overweight and obesity.

Outcomes	Risk of bias	Inconsistency	Indirectness	Imprecision	Publication bias	Number (INT/CON)	WMD (95% CI)	Quality of evidence
BF%	Serious^a^	Not serious	Serious^b^	Serious^c^	None	129/129	−0.72 (−1.11, −0.34)	⨁◯◯◯ Very low
BMI	Serious^a^	Not serious	Serious^b^	Not serious	None	238/240	−0.35 (−0.55, −0.14)	⨁⨁◯◯ Low
HC	Serious^a^	Not serious	Serious^b^	Very serious^c^, ^d^	None	60/61	−0.21 (−1.80, 1.39)	⨁◯◯◯ Very low
WC	Serious^a^	Not serious	Serious^b^	Very serious^c^, ^d^	Publication bias strongly suspected^e^	81/81	−0.30 (−2.06, 1.46)	⨁◯◯◯ Very low
Weight	Serious^a^	Not serious	Serious^b^	Not serious	Publication bias strongly suspected^e^	282/282	−1.41 (−2.19, −0.64)	⨁◯◯◯ Very low
WHR	Serious^a^	Not serious	Serious^b^	Serious^c^	None	64/62	−0.01 (−0.01, −0.01)	⨁◯◯◯ Very low
FBG	Very serious^a^	Serious^f^	Serious^b^	Very serious^c^, ^d^	None	125/126	−1.71 (−5.21, 1.79)	⨁◯◯◯ Very low
HOMA‐IR	Very serious^a^	Serious^f^	Very serious^b^	Serious^c^	Publication bias strongly suspected^e^	116/119	−0.16 (−0.29, −0.04)	⨁◯◯◯ Very low
Insulin	Very serious^a^	Not serious	Serious^b^	Serious^c^	None	125/126	−1.00 (−1.34, −0.66)	⨁◯◯◯ Very low
HDL‐C	Serious^a^	Not serious	Serious^b^	Very serious^c^, ^d^	None	189/189	1.54 (−0.24, 3.32)	⨁◯◯◯ Very low
LDL‐C	Serious^a^	Serious^f^	Serious^b^	Serious^c^	None	181/181	−5.73 (−8.77, −2.70)	⨁◯◯◯ Very low
TC	Serious^a^	Not serious	Serious^b^	Serious^c^	None	181/181	−6.62 (−10.18, −3.05)	⨁◯◯◯ Very low
TG	Serious^a^	Not serious	Serious^b^	Serious^c^	None	181/181	−3.24 (−6.25, −0.23)	⨁◯◯◯ Very low
ALP	Very serious^a^	Very serious^f^	Serious^b^	Extremely serious^c^, ^d^	None	41/40	−15.28 (−45.57, 15.01)	⨁◯◯◯ Very low
ALT	Very serious^a^	Very serious^f^	Serious^b^	Serious^c^	Publication bias strongly suspected^e^	81/80	−5.22 (−8.78, −1.65)	⨁◯◯◯ Very low
AST	Very serious^a^	Not serious	Serious^b^	Serious^c^	None	81/80	−3.45 (−5.00, −1.90)	⨁◯◯◯ Very low
CAT	Very serious^a^	Very serious^f^	Serious^b^	Serious^c^	None	69/72	19.15 (0.44, 37.85)	⨁◯◯◯ Very low
GPx	Very serious^a^	Very serious^f^	Serious^b^	Very serious^c^, ^d^	None	89/92	−3.30 (−11.71, 5.10)	⨁◯◯◯ Very low
MDA	Very serious^a^	Very serious^f^	Serious^b^	Very serious^c^, ^d^	None	110/111	−1.15 (−2.57, 0.26)	⨁◯◯◯ Very low
SOD	Very serious^a^	Very serious^f^	Serious^b^	Serious^c^	None	42/42	20.53 (15.03, 26.02)	⨁◯◯◯ Very low
TAC	Very serious^a^	Very serious^f^	Serious^b^	Very serious^c^, ^d^	None	62/62	−0.09 (−1.35, 1.17)	⨁◯◯◯ Very low
Leptin	Very serious^a^	Very serious^f^	Serious^b^	Serious^c^	None	30/30	−0.66 (−1.24, −0.08)	⨁◯◯◯ Very low

Abbreviations: ALP, Alkaline Phosphatase; ALT, Alanine Aminotransferase; AST, Aspartate Aminotransferase; BF%, Body Fat Percentage; BMI, Body Mass Index; CAT, Catalase; CI, Confidence Interval; CON, Control Group; FBG, Fasting Blood Glucose; GPx, Glutathione Peroxidase; HC, Hip Circumference; HDL‐C, High‐Density Lipoprotein Cholesterol; HOMA‐IR, Homeostatic Model Assessment of Insulin Resistance; INT, Intervention Group; LDL‐C, Low‐Density Lipoprotein Cholesterol; MDA, Malondialdehyde; SOD, Superoxide Dismutase; TAC, Total Antioxidant Capacity; TC, Total Cholesterol; TG, Triglycerides; WC, Waist Circumference; WHR, Waist‐to‐Hip Ratio; WMD, Weighted Mean Difference.

^a^ Downgraded since more than 50% of the participants were from high‐risk bias studies.

^b^ Downgraded for indirectness in the country.

^c^ Downgraded since the participants included were fewer than 400 persons.

^d^ Downgraded since the 95% CI crosses the threshold of interest.

^e^ Publication Bias was detected through Egger and Begg's test. (*p*‐value < 0.05).

^f^ The *I* ^2^ value was > 50% (or heterogeneity among the studies was high).

## 4 Discussion

The present meta‐analysis provides a focused synthesis of evidence regarding the effects of *Chlorella* supplementation on cardiometabolic risk factors specifically in individuals with overweight or obesity. *Chlorella* supplementation has been shown to yield significant beneficial effects on BF%, weight, and BMI. The genus *Chlorella* encompasses multiple taxonomically distinct species, most notably *C. vulgaris* and *C. pyrenoidosa* , which exhibit considerable interspecies variation in bioactive constituent profiles, including differences in chlorophyll content, carotenoid composition, polyunsaturated fatty acid distribution, and antioxidant capacity. These compositional disparities may theoretically translate into differential therapeutic efficacy across metabolic outcomes; however, our subgroup analysis stratified by *Chlorella* species revealed no statistically significant heterogeneity in cardiometabolic effects between *C. vulgaris* and *C. pyrenoidosa* , suggesting that the observed beneficial effects may be attributable to conserved bioactive mechanisms common across these species rather than species‐specific phytochemical profiles.

Subgroup analysis further indicated that reduced BF%, weight, and BMI are more pronounced in healthy adults. This amplified effect in healthy subjects may be attributed to more favorable metabolic responses to the bioactive compounds of *Chlorella*, which may influence lipid metabolism and fat oxidation processes (Huffman et al. 2014). In contrast, the presence of metabolic disorders may attenuate the metabolic response rate and diminish these benefits (Monfort‐Pires and Ferreira 2017). Monfort‐Pires et al. reported that subjects without metabolic syndrome were more responsive to a modified diet enriched with unsaturated fatty acids and fibers to improve HDL‐C and tumor necrosis factor‐alpha (TNF‐α) levels (Monfort‐Pires and Ferreira 2017).

Additionally, our findings suggest that female subjects may experience more beneficial effects in terms of BF% and BMI reductions. This effect may be attributed to hormonal differences, as well as the higher proportion of subcutaneous fat in women compared to men (Power and Schulkin 2008). A probable mechanism for this finding could be that subcutaneous fat shows more responsiveness to dietary interventions (Merlotti et al. 2017; Yarizadeh et al. 2021), potentially enhancing the effectiveness of *Chlorella* supplementation in females. While a significant weight reduction was observed in the male group, suggesting a gender‐specific response. This weight reduction in male individuals may be attributed to an overall decrease in body mass, potentially including losses in fat, muscle, and water weight. *Chlorella vulgaris* was also more effective than *Chlorella pyrenoidosa* in reducing BF% and BMI in short‐term supplementation (≤ 8 weeks) with a lower dosage of *Chlorella* (< 1500 mg).

*Chlorella* supplementation effectively reduced HOMA‐IR and insulin levels significantly. Subgroup analyses revealed that men experience greater benefits from long‐term *Chlorella* administration (over 8 weeks), particularly in terms of reducing FBG and insulin levels. Since men have a high basal metabolic rate (BMR) and a greater lean body mass, which contributes to increased glucose uptake (Maciak et al. 2020), it seems to enhance the beneficial effects of *Chlorella* supplementation in men. In an animal study, mice with a higher BMR showed increased expression of mTOR, which plays a role in suppressing the development of T2DM (Maciak et al. 2020). Also, testosterone has been shown to improve insulin sensitivity (Corona et al. 2011). Moreover, long‐term supplementation allows for a more gradual and sustained improvement in metabolic health. Furthermore, it has been shown that higher doses (≥ 1500 g) of *Chlorella vulgaris* could significantly reduce insulin levels in healthy individuals with obesity (BMI ≥ 30 kg/m^2^).

Pooled effect sizes revealed a significant impact of *Chlorella* on TC, LDL‐C, and TG levels. Several underlying mechanisms have been proposed for this finding. *Chlorella* with antioxidant properties may alleviate oxidative stress (Tsamesidis et al. 2024; Abdel‐Tawwab et al. 2023) and improve lipid metabolism (Lee et al. 2008). A study illustrated that a 10% *Chlorella*‐containing diet improved serum TC, TG, and liver total lipid and TC in rats (Lee et al. 2008). However, subgroup analysis indicated that healthy men with obesity under 40 years of age experience greater benefits from *Chlorella* supplementation, particularly in terms of reductions in TC, LDL‐C, and TG levels. Consistent with this finding, a previous meta‐analysis demonstrated a significant lipid‐lowering effect of *Chlorella* on TC levels (Sherafati et al. 2022). Similarly, in another study, participants in the *Chlorella*‐supplemented group demonstrated a significant decrease in TC and TG levels compared to the control group (Ryu et al. 2014).

Furthermore, supplementation with *Chlorella* species (*vulgaris* and *pyrenoidosa*) over 8 weeks reduced total TC and LDL‐C levels. Notably, the male group exhibited a significant effect of *Chlorella vulgaris* on HDL‐C levels with long‐term supplementation. Whereas, Sherafati et al. reported a non‐gender‐specific effect of *Chlorella*, with a pooled analysis of 10 studies showing no significant difference between males and females (Sherafati et al. 2022).

The combined effect size of the included studies illustrated that *Chlorella* could significantly reduce ALT and AST levels. The significant reduction in ALT and AST levels following *Chlorella* supplementation suggests a potential hepatoprotective effect. Previous meta‐analyses reported similar results in terms of the hepatoprotective properties of *Chlorella* (Yarmohammadi et al. 2021b). Although the exact mechanism underlying *Chlorella*'s liver‐protective effects has not yet been fully clarified, evidence suggests that its lipid‐lowering properties, enhancement of insulin sensitivity, and ability to scavenge free radicals generated by various oxidative processes play a key role (Ebrahimi‐Mameghani et al. 2017, 2014a; Chitsaz et al. 2016).

The current meta‐analysis revealed that *Chlorella* could promote an anti‐oxidative state by enhancing CAT activity and SOD levels. This finding suggests that *Chlorella* boosts the body's natural defense mechanisms against oxidative stress (Sikiru et al. 2019). Studies have shown that *Chlorella* may help neutralize free radicals and reactive oxygen species (ROS), highlighting its potential role in protecting against chronic diseases (Sikiru et al. 2019; Napolitano et al. 2020). Several studies have reported the antioxidative properties of *Chlorella*, including its ability to increase the levels of SOD and CAT, thus enhancing the body's overall antioxidant capacity (Abdel‐Karim et al. 2019; Mtaki et al. 2020). These findings support the notion that *Chlorella* could be a valuable supplement for reducing oxidative stress and promoting health. Subgroup analyses showed that an extended supplementation period with a higher dosage reduces MDA levels significantly. A reduction in MDA levels indicates a decrease in oxidative damage, implying that *Chlorella* supplementation may help protect cells from oxidative stress. The significant decrease in leptin levels following *Chlorella* supplementation suggests that *Chlorella* may be involved in metabolism. Also, decreased leptin may indicate decreased BF% and improved leptin sensitivity. Overall, it demonstrates that *Chlorella* supports beneficial effects on metabolic health.

### 4.1 Safety and Tolerability

Our meta‐analytic findings indicate favorable cardiometabolic outcomes associated with *Chlorella* supplementation in individuals with excess weight; however, rigorous clinical assessment necessitates critical examination of safety parameters. A substantial methodological limitation across the included trials was the paucity of systematic adverse event documentation, with few investigations implementing structured safety surveillance protocols. Evidence from the broader scientific literature suggests that *Chlorella* supplementation within conventional dosage ranges demonstrates acceptable tolerability profiles, with gastrointestinal manifestations (including nausea, abdominal distension, and diarrhea) representing the predominant adverse reactions, principally attributable to the substantial dietary fiber composition of *Chlorella* (Bito et al. 2020b; Panahi et al. 2016). Nonetheless, several significant safety considerations merit clinical attention. The bioaccumulation potential of microalgae presents substantial risks for heavy metal contamination (lead, cadmium, mercury, arsenic) and cyanotoxin accumulation when cultivated in suboptimal environmental conditions (Rzymski et al. 2015; Heussner et al. 2012). Prolonged ingestion of contaminated preparations may precipitate hepatotoxicity and additional severe pathological sequelae, emphasizing the critical necessity of procuring supplements from manufacturers adhering to stringent quality assurance protocols, including independent third‐party analytical verification (Dietrich and Hoeger 2005).

The scarcity of systematic safety surveillance across the majority of included investigations precludes definitive determination of adverse event incidence within the study populations examined. Although current evidence indicates reasonable safety margins when utilizing appropriately sourced, high‐quality preparations, the cardiometabolic improvements identified in our analysis must be judiciously evaluated against potential contamination hazards, pharmacological interactions, and existing limitations in safety evidence when formulating individualized patient recommendations.

### 4.2 Clinical Implications

The results of this meta‐analysis suggest that *Chlorella* may hold significant promise as an adjunctive strategy in the management of overweight and obesity. As global rates of obesity continue to escalate, and with them the burden of metabolic disorders such as T2DM, dyslipidemia, and nonalcoholic fatty liver disease, there is an urgent demand for safe, affordable, and broadly accessible interventions. Within this context, *Chlorella* presents as a compelling candidate due to its favorable safety profile, nutrient density, and potential for multi‐system benefits.

Beyond its potential to reduce weight and fat accumulation, *Chlorella* supplementation modulates several key biological processes relevant to metabolic health. These include improvements in insulin sensitivity, lipid metabolism, liver function, systemic inflammation, and oxidative stress. Such a broad spectrum of effects suggests that *Chlorella* may exert its influence through pathways beyond simple caloric reduction, potentially interacting with metabolic, hormonal, and inflammatory signaling cascades. These mechanistic insights support its classification not merely as a dietary supplement, but as a candidate functional food with therapeutic relevance. Clinically, *Chlorella* could be a valuable adjunct to established lifestyle‐based interventions, including dietary modifications, physical activity, and behavioral therapy. It may be especially advantageous for individuals who are either unable or unwilling to initiate pharmacologic treatment or those seeking integrative health approaches. In low‐resource settings, its low cost and ease of administration may enhance population‐level accessibility and adherence.

Nevertheless, practical implementation requires careful attention to formulation quality, dosage, and individual variation in response. Clinicians should exercise discernment when recommending *Chlorella*, tailoring its use to each patient's metabolic profile, comorbid conditions, and overall treatment goals. Until further evidence provides standardized guidelines, *Chlorella* should be integrated as a complementary, rather than primary, strategy within comprehensive obesity management plans.

### 4.3 Strengths and Limitations

This meta‐analysis represents the most comprehensive and methodologically robust synthesis to date examining the effects of *Chlorella vulgaris* supplementation in individuals with overweight and obesity. A principal strength of this review lies in its strict adherence to internationally accepted methodological standards. The study protocol was pre‐registered in PROSPERO, and the entire process conformed to the PRISMA 2020 guidelines, thereby ensuring transparency, replicability, and a systematic approach to evidence aggregation. The literature search was deliberately exhaustive, encompassing five major databases and gray literature sources, and was conducted without language or publication year restrictions. This breadth minimized selection bias and enhanced the completeness of evidence capture.

Another notable strength is the rigorous application of the GRADE framework to assess the certainty of evidence across a wide range of anthropometric, metabolic, hepatic, and oxidative stress markers. This approach enabled a more granular and clinically relevant evaluation of the available data. Additionally, the inclusion of subgroup analyses, meta‐regression, and dose–response modeling allowed for an exploration of potential effect modifiers such as participant age, sex, dosage, duration of supplementation, and supplement formulation, yielding valuable insights into factors that may influence the efficacy of *Chlorella* in distinct subpopulations.

Importantly, this review is one of the first to focus exclusively on populations with overweight and obesity, a group at elevated risk for cardiometabolic disorders but underrepresented in earlier analyses. Previous reviews often grouped heterogeneous populations or emphasized limited outcomes, whereas the current study adopted a more holistic perspective, incorporating a diverse array of clinically significant parameters such as insulin resistance, lipid metabolism, liver enzymes, inflammation, and oxidative stress. Moreover, by restricting inclusion to trials with clearly described intervention and control arms, the internal validity of the findings was further strengthened. It is worth highlighting that a key strength of this meta‐analysis lies in its comprehensive scope (it is the first to systematically evaluate the impact of *Chlorella* across a broad range of health outcomes, specifically in individuals with obesity and overweight).

Nevertheless, several limitations should be acknowledged. The methodological quality of many included studies was modest, with recurring concerns related to inadequate blinding, unclear allocation concealment, and incomplete outcome reporting. These weaknesses introduce the potential for performance and detection biases, which may have affected the reliability of the reported outcomes. The overall certainty of the evidence, as assessed by the GRADE framework, was rated as low to very low for most outcomes, limiting confidence in the observed effects. Inconsistencies in the standardization of *Chlorella* preparations (specifically regarding quantifying active components such as chlorophylls, carotenoids, and polysaccharides) also limit the ability to draw conclusions about which bioactive constituents are most influential. The geographical concentration of studies, with the majority conducted in Iran and very few from other parts of the world, further restricts the generalizability of the findings. The lack of representation from North America, Africa, and parts of Europe limits the applicability of the results to populations with different dietary patterns, cultural practices, and genetic backgrounds.

Additionally, the relatively short durations of most interventions (1–12 weeks) preclude conclusions regarding *Chlorella* supplementation's long‐term sustainability and safety. According to the GRADE quality assessment, one critical issue is the generally low quality of the included studies, which may weaken the reliability of the aggregated findings. Furthermore, the variability in participants' underlying health conditions across studies introduces another source of potential heterogeneity, making it difficult to attribute observed effects solely to *Chlorella* supplementation.

### 4.4 Future Research Directions

While the current analysis reinforces the therapeutic potential of *Chlorella* in obesity management, it also illuminates several areas where further research is urgently needed. A critical priority for future investigations is the execution of long‐term studies. These high‐quality trials are adequately powered and include participants from diverse ethnic, geographic, and clinical backgrounds. Such trials would enhance the external validity of findings and facilitate the development of population‐specific recommendations. Standardization of intervention protocols is also essential. Future studies should use well‐characterized *Chlorella* formulations with verified concentrations of bioactive compounds to allow for meaningful comparison across trials. The current heterogeneity in supplement forms (ranging from dried powders to capsules and water‐based extracts) complicates the interpretation of efficacy and the identification of dose–response relationships.

Further mechanistic exploration is warranted to elucidate the biological pathways through which *Chlorella* exerts its effects. Specifically, research should investigate its role in modulating adipose tissue biology, hormonal regulation, mitochondrial function, and inflammatory signaling. Employing emerging tools such as transcriptomics, metabolomics, and microbiome analysis could reveal personalized responses to supplementation and guide precision nutrition strategies. Longer intervention durations are necessary to evaluate the sustainability of metabolic improvements and to determine whether *Chlorella* supplementation influences long‐term outcomes such as type 2 diabetes onset, hepatic fibrosis progression, or cardiovascular events. Investigating its potential interactions with standard pharmacotherapies and other dietary supplements will also be essential for defining its role in multi‐modal treatment paradigms.

Incorporating *Chlorella* into functional food products or fortified dietary interventions offers a promising avenue for translation into real‐world settings. Research focused on formulation development, sensory acceptability, and adherence could facilitate its integration into public health strategies to reduce the burden of obesity‐related diseases.

## 5 Conclusion

In conclusion, this study demonstrates that *Chlorella* supplementation may improve key obesity‐related outcomes, including anthropometric indices, IR, lipid profile, liver enzyme levels, antioxidant capacity, and leptin concentrations. Subgroup analyses further suggest greater benefits with longer intervention durations and among male participants. While methodological limitations exist, the consistency of effects across multiple domains highlights *Chlorella*'s potential as a supportive strategy in the dietary management of overweight and obesity. Further well‐designed, long‐term trials are needed to confirm and extend these findings.

## Author Contributions

Conceptualization: Ali Jafari. Data curation: Ali Jafari, Helia Mardani, Mahsa Mahmoudinezhad. Formal analysis: Ali Jafari, Vali Musazadeh, Mohammad Sharifi. Investigation: Ali Jafari, Helia Mardani, Mohammad Amin Karimi, Mahsa Mahmoudinezhad, Vali Musazadeh, Mohammad Sharifi. Methodology: Ali Jafari, Vali Musazadeh, Mohammad Sharifi. Project administration: Ali Jafari, Vali Musazadeh, Mohammad Sharifi. Software: Ali Jafari, Helia Mardani, Vali Musazadeh, Mohammad Sharifi. Visualization: Mohammad Sharifi. Supervision: Ali Jafari, Vali Musazadeh, Mohammad Sharifi. Validation: Ali Jafari, Helia Mardani, Mohammad Amin Karimi, Vali Musazadeh, Mohammad Sharifi. Writing – original draft: Ali Jafari, Helia Mardani, Mohammad Amin Karimi. Writing – review and editing: Ali Jafari, Mahsa Mahmoudinezhad, Vali Musazadeh, Mohammad Sharifi.

## Funding

The authors have nothing to report.

## Ethics Statement

As a systematic review and meta‐analysis, this manuscript did not necessitate review by our institutional clinical ethics committee.

## Consent

The authors have nothing to report.

## Conflicts of Interest

Ali Jafari holds an editorial position at *Systematic Reviews*. All other authors of this publication declare that they have no affiliations with, or involvement in, any organization or entity with a financial interest (including honoraria, educational grants, participation in speakers' bureaus, memberships, employment, consultancies, stock ownership or other equity interests, expert testimony, or patent‐licensing arrangements) or a non‐financial interest (such as personal or professional relationships, affiliations, knowledge, or beliefs) relevant to the subject matter or materials discussed in this manuscript.

## Supporting information


**Table S1:** Search strategy to find potential eligible randomized controlled trials (November 2024).
**Table S2:** A summary of excluded articles after full text review.
**Figure S1:** Funnel plot assessing potential publication bias in the meta‐analysis of *Chlorella* supplementation and cardiovascular disease risk factors in individuals with overweight or obesity (a: body fat percentage, b: body mass index, c: weight, d: fasting blood glucose, e: homeostatic model assessment of insulin resistance, f: insulin, g: high‐density lipoprotein cholesterol, h: low‐density lipoprotein cholesterol, i: total cholesterol, j: triglycerides, k: malondialdehyde).
**Figure S2:** Non‐linear dose–response relations between *Chlorella* dosage (mg/d) and cardiovascular disease risk factors in individuals with overweight or obesity (a: body fat percentage, b: body mass index, c: weight, d: fasting blood glucose, e: homeostatic model assessment of insulin resistance, f: insulin, g: high‐density lipoprotein cholesterol, h: low‐density lipoprotein cholesterol, i: total cholesterol, j: triglycerides, k: malondialdehyde).
**Figure S3:** Non‐linear dose–response relations between duration of intervention and cardiovascular disease risk factors in individuals with overweight or obesity (a: body fat percentage, b: body mass index, c: weight, d: fasting blood glucose, e: homeostatic model assessment of insulin resistance, f: insulin, g: high‐density lipoprotein cholesterol, h: low‐density lipoprotein cholesterol, i: total cholesterol, j: triglycerides, k: malondialdehyde).
**Figure S4:** Random‐effects meta‐regression plots of the association between *Chlorella* dosage (mg/d) and cardiovascular disease risk factors in individuals with overweight or obesity (a: body fat percentage, b: body mass index, c: weight, d: fasting blood glucose, e: homeostatic model assessment of insulin resistance, f: insulin, g: high‐density lipoprotein cholesterol, h: low‐density lipoprotein cholesterol, i: total cholesterol, j: triglycerides, k: malondialdehyde).
**Figure S5:** Random‐effects meta‐regression plots of the association between duration of intervention and cardiovascular disease risk factors in individuals with overweight or obesity (a: body fat percentage, b: body mass index, c: weight, d: fasting blood glucose, e: homeostatic model assessment of insulin resistance, f: insulin, g: high‐density lipoprotein cholesterol, h: low‐density lipoprotein cholesterol, i: total cholesterol, j: triglycerides, k: malondialdehyde).

## Data Availability

All relevant data are provided within the manuscript and Supporting Information. Additionally, the data analyzed for this study are available upon request from the corresponding author.
