# Supplementary material for: Does Chlorella Supplementation Improve Adiposity, Metabolic Dysfunction, and Oxidative Stress in Individuals With Excess Weight? A Systematic Review and Meta‐Analysis
Source: Food Sci Nutr. 2026 Apr 22;14(4):e71715. doi: 10.1002/fsn3.71715 (PMC13100651; doi:10.1002/fsn3.71715)
Supplement: Supplementary file 1 — Table S1: Search strategy to find potential eligible randomized controlled trials (November 2024). Table S2: A summary of excluded articles after full text review. Figure S1: Funnel plot assessing potential publication bias in the meta‐analysis of Chlorella supplementation and cardiovascular disease risk factors in individuals with overweight or obesity (a: body fat percentage, b: body mass index, c: weight, d: fasting blood glucose, e: homeostatic model assessment of insulin resistance, f: insulin, g: high‐density lipoprotein cholesterol, h: low‐density lipoprotein cholesterol, i: total cholesterol, j: triglycerides, k: malondialdehyde). Figure S2: Non‐linear dose–response relations between Chlorella dosage (mg/d) and cardiovascular disease risk factors in individuals with overweight or obesity (a: body fat percentage, b: body mass index, c: weight, d: fasting blood glucose, e: homeostatic model assessment of insulin resistance, f: insulin, g: high‐density lipoprotein cholesterol, h: low‐density lipoprotein cholesterol, i: total cholesterol, j: triglycerides, k: malondialdehyde). Figure S3: Non‐linear dose–response relations between duration of intervention and cardiovascular disease risk factors in individuals with overweight or obesity (a: body fat percentage, b: body mass index, c: weight, d: fasting blood glucose, e: homeostatic model assessment of insulin resistance, f: insulin, g: high‐density lipoprotein cholesterol, h: low‐density lipoprotein cholesterol, i: total cholesterol, j: triglycerides, k: malondialdehyde). Figure S4: Random‐effects meta‐regression plots of the association between Chlorella dosage (mg/d) and cardiovascular disease risk factors in individuals with overweight or obesity (a: body fat percentage, b: body mass index, c: weight, d: fasting blood glucose, e: homeostatic model assessment of insulin resistance, f: insulin, g: high‐density lipoprotein cholesterol, h: low‐density lipoprotein cholesterol, i: total cholesterol, j: triglycerides, [file FSN3-14-e71715-s001.docx]

**Does Chlorella Supplementation Improve Adiposity, Metabolic Dysfunction, and Oxidative Stress in Individuals with Excess Weight? A Systematic Review and Meta-Analysis and Meta-Analysis**

**Ali Jafari^1,2^, Helia Mardani^3^,** **Mahsa Mahmoudinezhad^4,5^, Mohammad Amin Karimi^6^, Vali Musazadeh^7,8*^, Mohammad Sharifi^9*^**

^1^Student Research Committee, Department of Community Nutrition, Faculty of Nutrition Sciences and Food Technology, National Nutrition and Food Technology Research Institute, Shahid Beheshti University of Medical Sciences, Tehran, Iran

^2^Systematic Review and Meta-analysis Expert Group (SRMEG), Universal Scientific Education and Research Network (USERN), Tehran, Iran

^3^Students' Scientific Research Center (SSRC), Tehran University of Medical Sciences, Tehran, Iran

^4^Food and Beverages Safety Research Center, Urmia University of Medical Sciences, Urmia, Iran.

^5^Student Research Committee, Urmia University of Medical Sciences, Urmia, Iran

^6^School of Medicine, Shahid Beheshti University of Medical Sciences, Tehran, Iran

^7^Student Research Committee, School of Public Health, Iran University of Medical Sciences, Tehran, Iran

^8^Department of Nutrition, School of Public Health, Iran University of Medical Sciences, Tehran, Iran

^9^Department of Nutrition, Food Sciences and Clinical Biochemistry, School of Medicine, Social Determinants of Health Research Center, Gonabad University of Medical Science, Gonabad, Iran

Supplementary materials**:** Supplementary Tables 1-2, Supplementary Figure 1-5, and Supplementary References.

| **Supplementary Table 1.** Search strategy to find potential eligible randomised controlled trials (November 2024) | |
| --- | --- |
| **Groups** | **Descriptors** |
| Intervention | Chlorella OR Chlorellas OR microalgae OR C. vulgaris OR C. pyrenoidosa OR Chlorella pyrenoidosa |
| Population | Obesity OR obese OR overweight |
| Design | Intervention OR “Intervention Study” OR “Intervention Studies” OR “controlled trial” OR random* OR placebo OR “clinical trial” OR Trial OR “randomized controlled trial” OR “randomized clinical trial” OR RCT OR blinded OR “double blind” OR “double blinded” OR trial* OR “Pragmatic Clinical Trial” OR “Cross-Over Studies” OR “Cross-Over” OR “Cross-Over Study” OR parallel OR “parallel study” OR “parallel trial” |

**PubMed**

Number of localized studies: 19

|  | **Descriptors** | **Number of studies reached** |
| --- | --- | --- |
| #1 | "Chlorella"[Mesh] OR "Chlorella vulgaris"[Mesh] OR Chlorella[tiab] OR Chlorellas[tiab] OR microalgae[tiab] OR Chlorella pyrenoidosa[tiab] OR C. vulgaris[tiab] OR C. pyrenoidosa[tiab] | 21,635 |
| #2 | obesity[tiab] OR obese[tiab] OR overweight[tiab] | 439,658 |
| #3 | Intervention[Title/Abstract] OR "Intervention Study"[Title/Abstract] OR "Intervention Studies"[Title/Abstract] OR "controlled trial"[Title/Abstract] OR random*[Title/Abstract] OR placebo[Title/Abstract] OR "clinical trial"[Title/Abstract] OR Trial[Title/Abstract] OR "randomized controlled trial"[Title/Abstract] OR "randomized clinical trial"[Title/Abstract] OR RCT[Title/Abstract] OR blinded[Title/Abstract] OR "double blind"[Title/Abstract] OR "double blinded"[Title/Abstract] OR trial*[Title/Abstract] OR "Pragmatic Clinical Trial"[Title/Abstract] OR "Cross-Over Studies"[Title/Abstract] OR "Cross-Over"[Title/Abstract] OR "Cross-Over Study"[Title/Abstract] OR parallel[Title/Abstract] OR "parallel study"[Title/Abstract] OR "parallel trial"[Title/Abstract] OR "Clinical Trial" [Publication Type] OR "Randomized Controlled Trial" [Publication Type] OR "Controlled Clinical Trial" [Publication Type] OR "Random Allocation"[Mesh] OR "Randomized Controlled Trials as Topic"[Mesh] OR "Pragmatic Clinical Trial" [Publication Type] OR "Pragmatic Clinical Trials as Topic"[Mesh] OR "Double-Blind Method"[Mesh] OR "Single-Blind Method"[Mesh] OR "Cross-Over Studies"[Mesh] | 3,776,389 |
| #4 | #1 AND #2 AND #3 | 19 |

**Web of Science**

Number of localized studies: 63

|  | **Descriptors** | **Number of studies reached** |
| --- | --- | --- |
| #1 | TS=( Chlorella OR Chlorellas* OR microalgae OR C. vulgaris* OR C. pyrenoidosa* OR Chlorella pyrenoidosa* ) | 78,469 |
| #2 | TS=( Obesity OR obese OR overweight) | 628,586 |
| #3 | TS=(Intervention OR “Intervention Study” OR “Intervention Studies” OR “controlled trial” OR random* OR placebo OR “clinical trial” OR Trial OR “randomized controlled trial” OR “randomized clinical trial” OR RCT OR blinded OR “double blind” OR “double blinded” OR trial* OR “Pragmatic Clinical Trial” OR “Cross-Over Studies” OR “Cross-Over” OR “Cross-Over Study” OR parallel OR “parallel study” OR “parallel trial”) | 6,607,258 |
| #4 | #1 AND #2 AND #3 | 63 |

**Scopus**

Number of localized studies: 44

|  | **Descriptors** | **Number of studies reached** |
| --- | --- | --- |
| #1 | TITLE-ABS-KEY ( Chlorella OR Chlorellas* OR microalgae OR "C. vulgaris*" OR "C. pyrenoidosa*" OR "Chlorella pyrenoidosa*") | 59,652 |
| #2 | TITLE-ABS-KEY (Obesity OR obese OR overweight) | 699,309 |
| #3 | TITLE-ABS-KEY (intervention OR "Intervention Study" OR "Intervention Studies" OR "controlled trial" OR random* OR placebo OR "clinical trial" OR trial OR "randomized controlled trial" OR "randomized clinical trial" OR rct OR blinded OR "double blind" OR "double blinded" OR trial* OR "Pragmatic Clinical Trial" OR "Cross-Over Studies" OR "Cross-Over" OR "Cross-Over Study" OR parallel OR "parallel study" OR "parallel trial") | 8,174535 |
| #4 | #1 AND #2 AND #3 | 44 |

**Embase**

Number of localized studies: 48

|  | **Descriptors** | **Number of studies reached** |
| --- | --- | --- |
| #1 | 'Chlorella'/exp OR 'Chlorellas'/exp OR 'microalgae'/exp OR 'C. vulgaris'/exp OR 'C. pyrenoidosa'/exp OR 'Chlorella pyrenoidosa'/exp | 26,105 |
|  | 'Obesity'/exp OR 'obese'/exp OR 'overweight'/exp | 777,222 |
| #2 | 'randomized controlled trial'/exp OR 'randomized controlled trial (topic)'/exp OR 'pragmatic trial'/exp OR 'clinical trial'/exp OR 'clinical trial (topic)'/exp OR 'intervention study'/exp OR 'controlled study'/exp OR 'controlled clinical trial'/exp OR 'double blind procedure'/exp OR 'single blind procedure'/exp OR 'crossover procedure'/exp OR 'parallel design'/exp | 12,067,575 |
| #3 | #1 AND #2 AND #3 | 48 |

**Cochrane**

Number of localized studies: 32

Limits: TRIALS

|  | **Descriptors** | **Number of studies reached** |
| --- | --- | --- |
| #1 | (Chlorella OR Chlorellas* OR microalgae OR C. vulgaris OR C. pyrenoidosa OR (Chlorella NEXT pyrenoidosa)):ti,ab,kw | 733 |
| #2 | (Obesity OR obese OR overweight):ti,ab,kw | 62,904 |
| #3 | #1 AND #2 | 32 |

| **Supplementary Table 2.** A summary of excluded articles after full text review | |
| --- | --- |
| **Author, Year (Ref.)** | **Reason** |
| Merchant, 2001 (1) | Not interested outcome |
| Nakano, 2010 (2) | Not interested population |
| Kwak, 2012 (3) | Not interested population |
| Lee, 2012 (4) | Co-administration |
| Ebrahimi-Mameghani, 2014 (5) | Co-administration |
| Plakida, 2020 (6) | Not interested study design |
| Uchiyama-Tanaka, 2023 (7) | Not interested outcome |
| White, 2024 (8) | Not interested outcome |


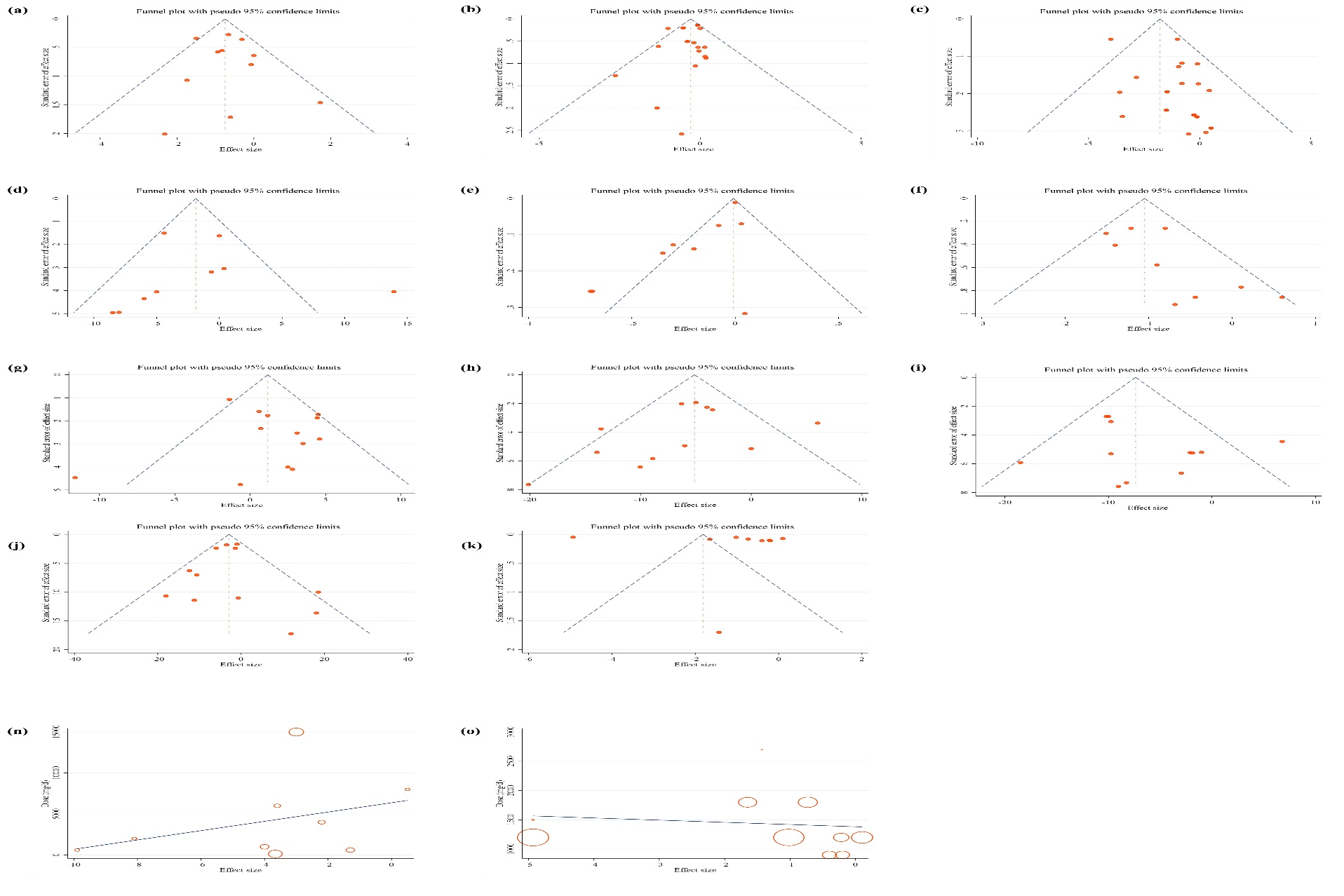


**Supplementary Figure 1.** Funnel plot assessing potential publication bias in the meta-analysis of chlorella supplementation and cardiovascular disease risk factors in individuals with overweight or obesity (a: body fat percentage, b: body mass index, c: weight, d: fasting blood glucose, e: homeostatic model assessment of insulin resistance, f: insulin, g: high-density lipoprotein cholesterol, h: low-density lipoprotein cholesterol, i: total cholesterol, j: triglycerides, k: malondialdehyde)


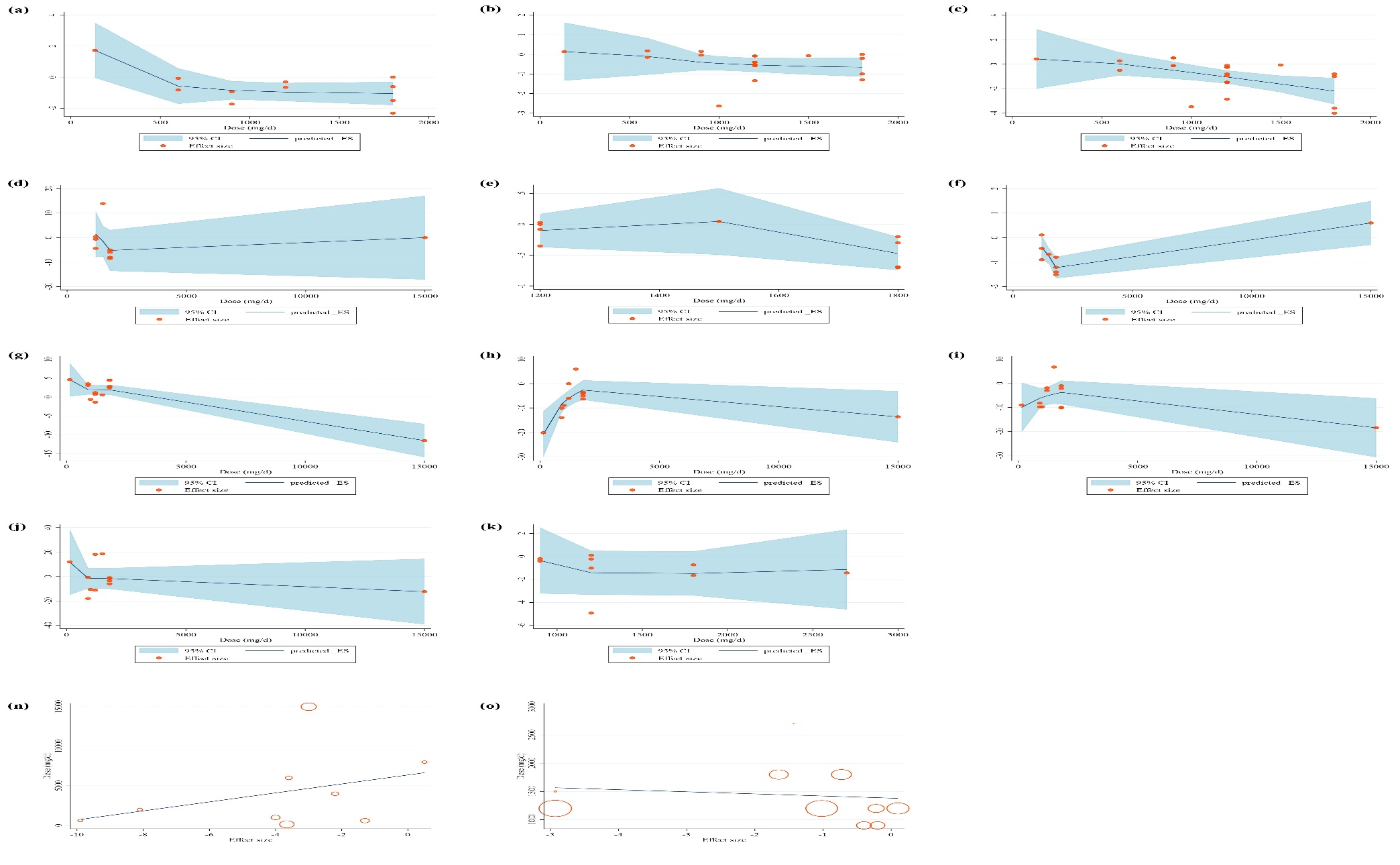


**Supplementary Figure 2.** Non-linear dose-response relations between chlorella dosage (mg/d) and cardiovascular disease risk factors in individuals with overweight or obesity (a: body fat percentage, b: body mass index, c: weight, d: fasting blood glucose, e: homeostatic model assessment of insulin resistance, f: insulin, g: high-density lipoprotein cholesterol, h: low-density lipoprotein cholesterol, i: total cholesterol, j: triglycerides, k: malondialdehyde)


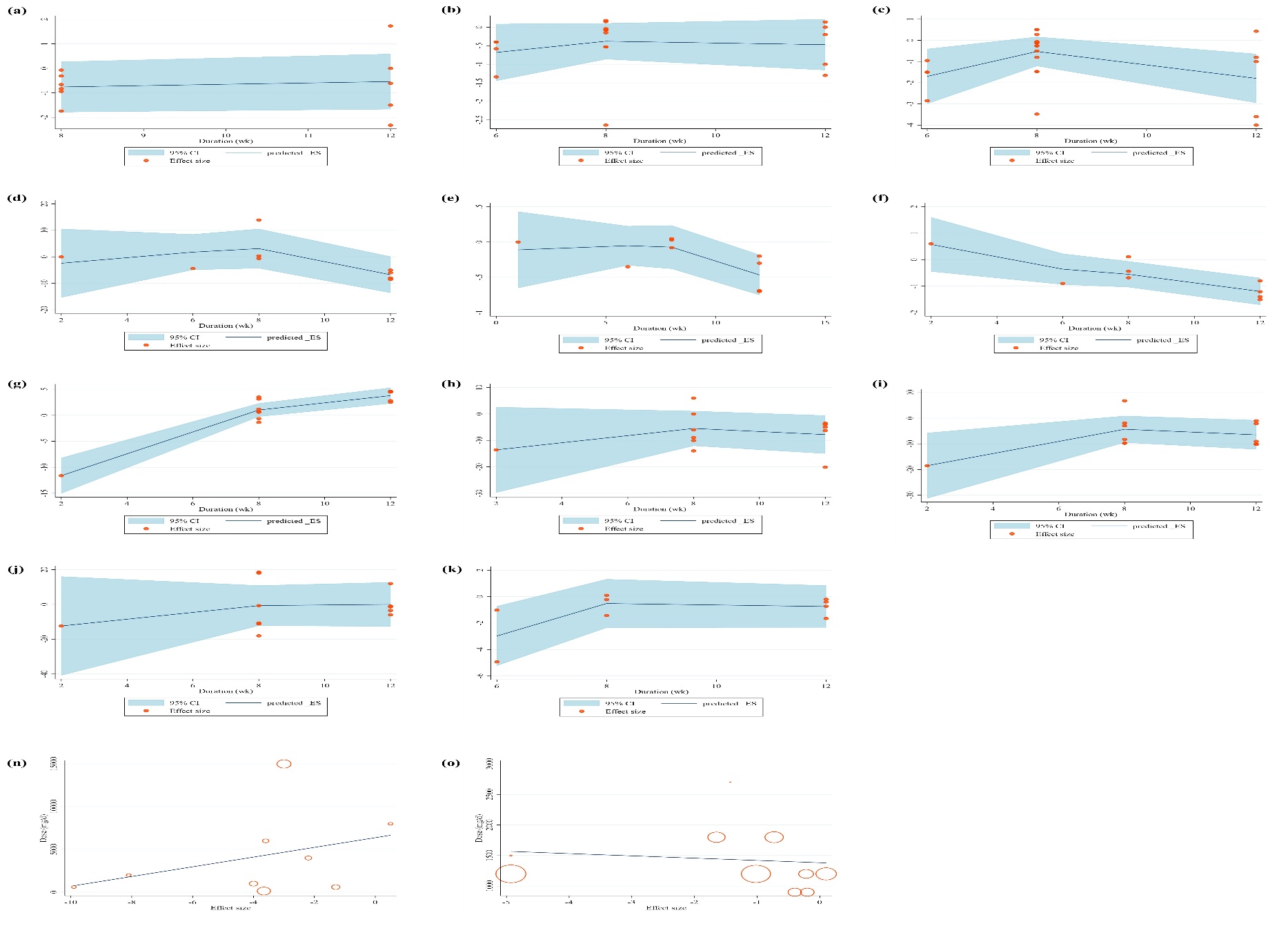


**Supplementary Figure 3.** Non-linear dose-response relations between duration of intervention and cardiovascular disease risk factors in individuals with overweight or obesity (a: body fat percentage, b: body mass index, c: weight, d: fasting blood glucose, e: homeostatic model assessment of insulin resistance, f: insulin, g: high-density lipoprotein cholesterol, h: low-density lipoprotein cholesterol, i: total cholesterol, j: triglycerides, k: malondialdehyde)


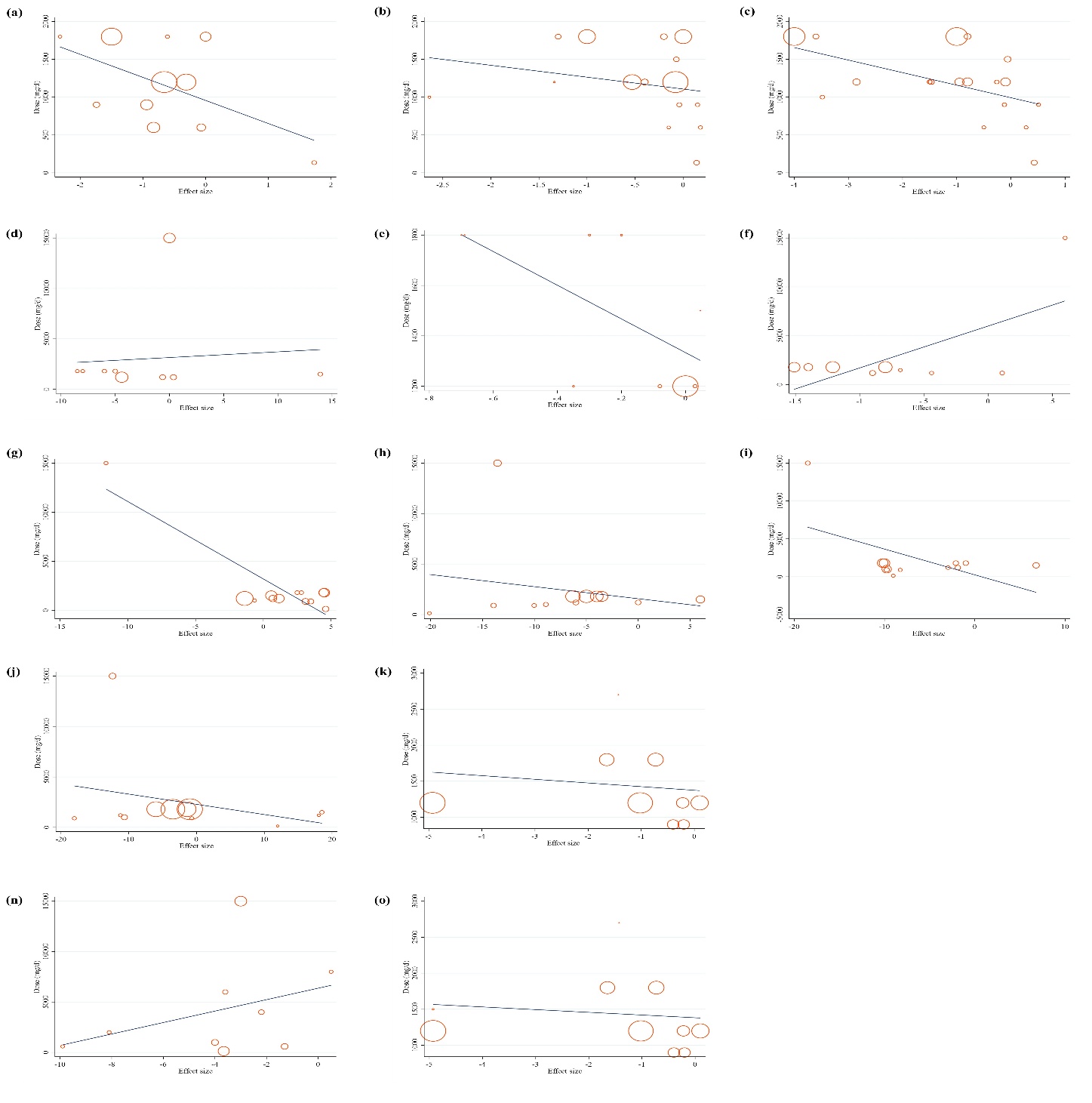


**Supplementary Figure 4.** Random-effects meta-regression plots of the association between chlorella dosage (mg/d) and cardiovascular disease risk factors in individuals with overweight or obesity (a: body fat percentage, b: body mass index, c: weight, d: fasting blood glucose, e: homeostatic model assessment of insulin resistance, f: insulin, g: high-density lipoprotein cholesterol, h: low-density lipoprotein cholesterol, i: total cholesterol, j: triglycerides, k: malondialdehyde)


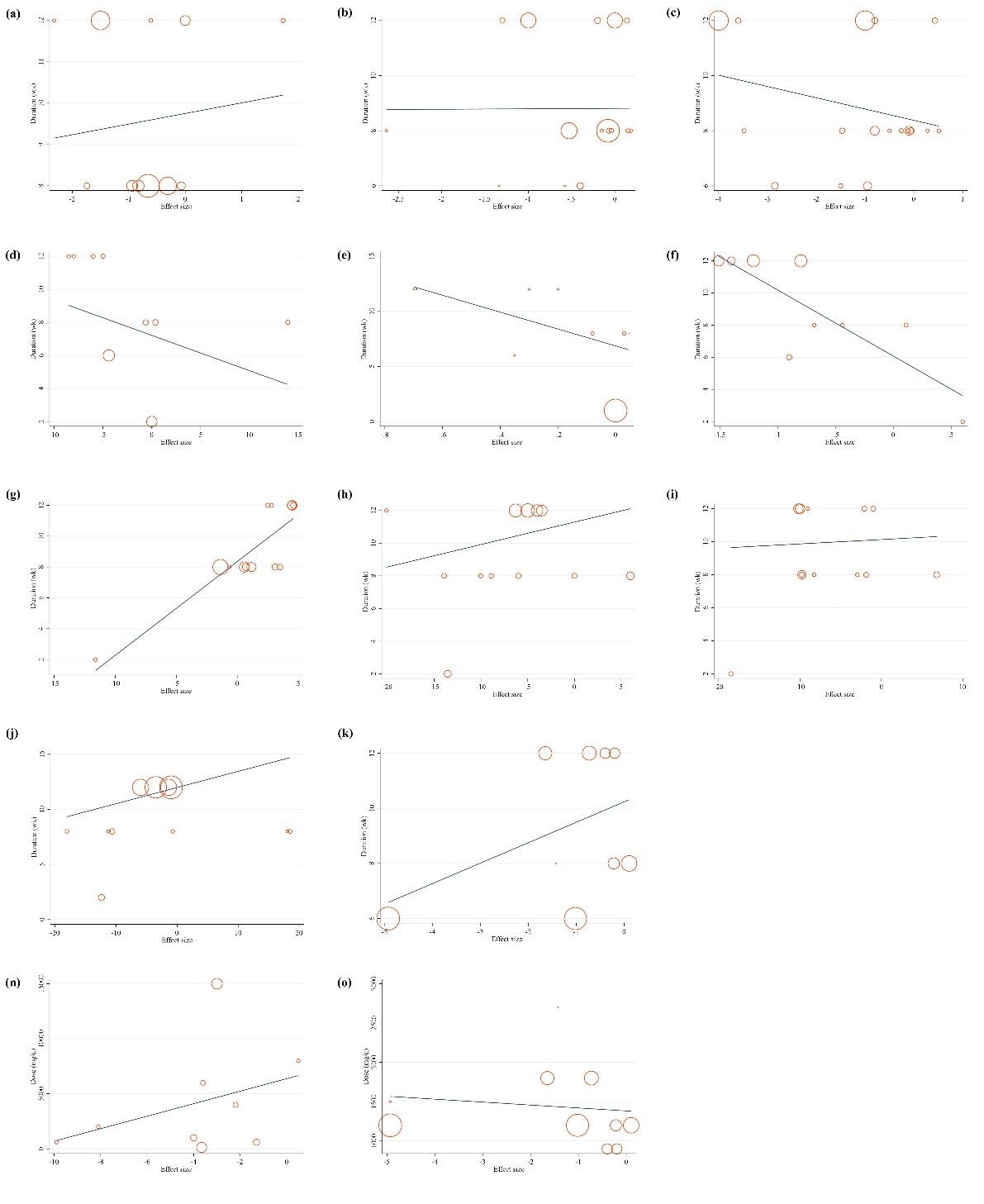


**Supplementary Figure 5.** Random-effects meta-regression plots of the association between duration of intervention and cardiovascular disease risk factors in individuals with overweight or obesity (a: body fat percentage, b: body mass index, c: weight, d: fasting blood glucose, e: homeostatic model assessment of insulin resistance, f: insulin, g: high-density lipoprotein cholesterol, h: low-density lipoprotein cholesterol, i: total cholesterol, j: triglycerides, k: malondialdehyde)

**Supplemental** **References**

1. Merchant RE, Andre CA, Wise CM. Nutritional supplementation with Chlorella pyrenoidosa for fibromyalgia syndrome: A double-blind, placebo-controlled, crossover study. Journal of Musculoskeletal Pain. 2001;9(4):37-54.

2. Nakano S, Takekoshi H, Nakano M. Chlorella pyrenoidosa supplementation reduces the risk of anemia, proteinuria and edema in pregnant women. Plant foods for human nutrition. 2010;65:25-30.

3. Kwak JH, Baek SH, Woo Y, Han JK, Kim BG, Kim OY, et al. Beneficial immunostimulatory effect of short-term Chlorella supplementation: enhancement of natural killer cell activity and early inflammatory response (randomized, double-blinded, placebo-controlled trial). Nutrition journal. 2012;11:1-8.

4. Lee I-T, Lee W-J, Tsai C-M, Su I-J, Yen H-T, Sheu WH. Combined extractives of red yeast rice, bitter gourd, chlorella, soy protein, and licorice improve total cholesterol, low-density lipoprotein cholesterol, and triglyceride in subjects with metabolic syndrome. Nutrition research. 2012;32(2):85-92.

5. Ebrahimi-Mameghani M, Aliashrafi S, Javadzadeh Y, AsghariJafarabadi M. The effect of Chlorella vulgaris supplementation on liver enzymes, serum glucose and lipid profile in patients with non-alcoholic fatty liver disease. Health promotion perspectives. 2014;4(1):107.

6. Plakida A, Yushkovskaya O, Filonenko A, Gushcha S. The Effect of the Aqueous Suspension of Chlorella Vulgaris on Functional Systems in Healthy People. Acta Balneologica. 2020;159(11).

7. Uchiyama-Tanaka Y, Okumura E, Fujishima M. Folate metabolism of Chlorella pyrenoidosa on subjects of MTHFR C677T polymorphism. Personalized Medicine Universe. 2023;12:33-7.

8. White H, Gurney T. Chlorella Supplementation Reduces Blood Lactate Concentration and Increases O2 Pulse during Submaximal and Maximal Cycling in Young Healthy Adults. Nutrients. 2024;16(5):697.
